# Evaluation of the Effects of Honey on Acute-Phase Deep Burn Wounds

**DOI:** 10.1155/2013/784959

**Published:** 2013-11-17

**Authors:** Yukari Nakajima, Kanae Mukai, Nasruddin, Emi Komatsu, Terumi Iuchi, Yukie Kitayama, Junko Sugama, Toshio Nakatani

**Affiliations:** Department of Clinical Nursing, Graduate Course of Nursing Science, Division of Health Sciences, Graduate School of Medical Science, Kanazawa University, 5-11-80 Kodatsuno, Kanazawa 920-0942, Japan

## Abstract

This study aimed to clarify the effects of honey on acute-phase deep burn wounds. Two deep burn wounds were created on mice which were divided into four groups: no treatment, silver sulfadiazine, manuka honey, and Japanese acacia honey. Wound sizes were calculated as expanded wound areas and sampled 30 minutes and 1–4 days after wounding for histological observation. The wound sections were subjected to hematoxylin and eosin and immunohistological staining to detect necrotic cells, apoptotic cells, neutrophils, and macrophages. The no treatment group formed a scar. The redness around the wound edges in the silver sulfadiazine group was the most intense. All groups exhibited increased wound areas after wounding. The proportions of necrotic cells and the numbers of neutrophils in the manuka and acacia honey groups were lower than those in the no treatment and silver sulfadiazine groups until day 3; however, there were no significant differences between all groups on day 4. These results show that honey treatment on deep burn wounds cannot prevent wound progression. Moreover, comparing our observations with those of Jackson, there are some differences between humans and animals in this regard, and the zone of hyperemia and its surrounding area fall into necrosis, which contributes to burn wound progression.

## 1. Introduction

Burns are dynamic injuries that are characterized by their area and depth. The extent of a burn wound can be calculated by at least 3 different methods: rule of nine, Lund and Browder chart, and palmar surface. The depth of a burn wound is mainly divided into 3 classes: superficial, partial thickness, and full thickness. The correct diagnosis of the depth often depends on a surgeon's experience and the timing of diagnosis because burn wounds progress after their formation [1]. 

 Jackson [2] described three concentric zones of burn wound from the intensity of burning and blood flow: the central zone of coagulation, the intermediate zone of stasis, and the outer zone of hyperemia. The white tissue in the zone of coagulation has the greatest direct damage from thermal trauma and is characterized by irreversible necrosis. Microscopically, the zone of coagulation demonstrates complete destruction of the subpapillary vasculature. The intermediate zone of stasis exhibits complete cessation of blood flow within 24 hours and tissue necrosis, and the red and white mottling of the zone of stasis turn white, so the continued tissue necrosis in the zone of stasis contributes to burn wound progression. The edematous, red tissue in the zone of hyperemia invariably recovers. Microscopically, the zone of hyperemia demonstrates almost complete loss of epidermis without apparent structural damage to the dermis, but capillary loops are patent in the dermis [2, 3].

In burn wounds, it has been revealed that honey decreased wound area in rats, had an antibacterial effect in rats [4], and promoted reepithelialization in pigs [5] compared with hydrofiber silver or silver sulfadiazine (SSD). These effects of honey resulted from its high osmolarity, enzymes, phytochemicals, acidity, and so on [6–9]. These previous studies demonstrated the effects of honey after burn wound progression. However, no study has focused on the effects of honey on acute-phase burn wounds, which is the condition before the zone of stasis in the wound falls into necrosis. However, Molan proposed that the anti-inflammatory action of honey would reduce the damage caused by free radicals that arise from inflammation and prevent further necrosis [10]. In the zone of stasis, prolonged inflammation dominated by neutrophils and macrophages leads to edema and hypoperfusion in tissue, neutrophils and macrophages produce oxygen free radicals by bacterial englobement, and the adherence of neutrophils to the venular endothelium results in microvascular compromise [3, 11, 12]. When phagocytes like neutrophils and macrophages are activated via the NADPH oxidase located in the cell membrane by bacteria, certain particles, or soluble stimulation, they produce a burst of free radicals. This serves as a significant protective mechanism in normovolemia by scavenging invading bacteria; however, with injury such as burn trauma, a burst of free radicals may exacerbate xanthine oxidase activity, producing overwhelming tissue damage like lipid peroxidation, protein oxidation, and oxidative DNA damage [11, 13, 14]. These phenomena by inflammation in the zone of stasis contribute to burn wound progression in acute-phase deep burn wounds, and, microscopically, cell necrosis and apoptosis occur in this zone [15, 16]. On the other hand, our previous study [17] using manuka honey revealed wound reduction in the inflammatory phase in full-thickness wounds because manuka honey shortened the inflammatory phase. Our previous study [18] using Japanese acacia honey also revealed this because of its anti-inflammatory action. Additionally, SSD is commonly used as an antibacterial agent, and its use on deep burn wounds is recommended in Japan [19]. Therefore, we hypothesize that manuka honey and Japanese acacia honey can prevent burn wound progression on acute-phase deep burn wounds better than no treatment or the application of SSD by decreasing the wound area, necrotic and apoptotic cells, inflammatory cells, and the inflammatory cytokine level. The aim of this study is thus to clarify the effects of honey on acute-phase deep burn wounds.

## 2. Materials and Methods

### 2.1. Animals

One hundred and twenty-six BALB/cCrSlc male mice aged 8 weeks (Sankyo Lab Service Corporation, Inc., Toyama, Japan) were used. They were caged individually in an air-conditioned room at 25.0 ± 2.0°C with light from 08:45 to 20:45 Water and laboratory chow were given freely. The experimental protocol and animal care were in accordance with the Guidelines for the Care and Use of Laboratory Animals of Kanazawa University, Japan (AP-122298).

### 2.2. Honey

We used two types of honey, New Zealand manuka (*Leptospermum scoparium*) honey and Japanese acacia (*Robinia pseudoacacia*) honey (Yamada Bee Farm, Okayama, Japan), because manuka honey is well known globally as a medicinal honey [9, 17] and Japanese acacia honey was the most effective for wound healing in our previous study [18].

### 2.3. Wounding

**Figure 1:**
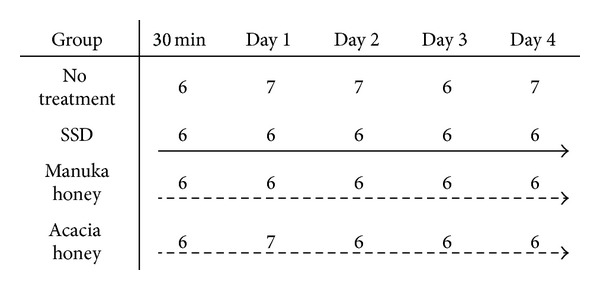
This figure shows the number of mice in each group and each day. The numbers 6 and 7 indicate the number of mice. (→) indicates daily treatment with 0.1 mL of SSD per wound and covering with gauze and bandages. (⇢) indicates the daily treatment with 0.2 mL of each honey per wound and covering with gauze and bandage.

In accordance with previous studies [17, 18], the mice were anesthetized with an intraperitoneal (IP) injection of pentobarbital sodium (0.05 mg/g weight), and the dorsum was shaved. Two circular (1.2 mm in diameter) full-thickness burn wounds on both sides of the dorsum of the mouse were made by applying a 10 g weight heated with boiling water (100°C) for 20 seconds. The mice were divided into four groups (Figure 1). Wounds in the experimental groups, manuka honey and acacia honey groups, were each treated with 0.2 mL of honey per wound. The wounds to which honey was applied were covered with gauze as a secondary dressing to prevent runoff and to absorb exudate that leaked from the surface of the wound. The gauze was changed, and all wounds were treated with honey every day. Mice in the honey groups were wrapped twice with a sticky bandage (Mesh pore tape; Nichiban, Tokyo, Japan). Meanwhile, wounds of the control groups, no treatment and SSD groups, were left untreated or treated with 0.1 mL of silver sulfadiazine (GEBEN cream 1%; Mitsubishi Tanabe Pharma, Osaka, Japan) per wound, respectively. The wounds to which SSD was applied were covered with gauze as a secondary dressing to maintain a moist environment. The gauze was changed, and all wounds were treated with SSD every day. Mice in the SSD group were wrapped twice with a sticky bandage (Mesh pore tape; Nichiban, Tokyo, Japan).

### 2.4. Macroscopic Observation

The day when wounds were made was designated as day 0. In a preliminary test, the wounds stopped increasing in size on day 4, so the observation period was set to last from day 0 to day 4 after wounding. Wound edges were traced on polypropylene sheets, and photographs were taken every day. The perimeter of the bottom of the weight used for wounding was also traced on polypropylene sheets. The traces of wound edges and the bottom of the weight on the sheets were captured with a scanner onto a personal computer using Adobe Photoshop Elements 7.0 (Adobe System Inc., Tokyo, Japan), and these areas were calculated using image analysis software Scion Image Beta 4.02 (Scion Corporation, Frederick, Maryland, USA). Then, the expanded wound areas were calculated by subtraction of the bottom area of the weight from the actual wound areas.

### 2.5. Preparation of Blood Serum Samples

After mice were euthanized by an IP injection of pentobarbital sodium, blood was taken from the heart into a 1.5 mL tube in order to measure the level of TNF-**α**; the blood was left to stand at room temperature for 30 minutes. It was then centrifuged at 1500 ×g for 15 minutes (4°C); finally, the serum was aliquoted and stored at −80°C until use.

### 2.6. Tissue Processing

The mice were euthanized by a massive IP injection of pentobarbital sodium 30 minutes and 1, 2, 3, and 4 days after wounding. The wounds and the surrounding intact skin were harvested, stapled onto transparent plastic sheets to prevent overcontraction of specimens, and fixed in 4% paraformaldehyde in 0.1 mol/L phosphate buffer (pH 7.4) for 17 hours. Specimens were dehydrated in an alcohol series, cleaned in xylene, and embedded in paraffin to prepare 5 *μ*m serial sections. Each serial section was stained with hematoxylin and eosin (H&E) and immunohistologically stained with anti-HMGB1 antibody (Abcam Japan, Tokyo, Japan) to detect necrotic cells, Cleaved Caspase-3 antibody to detect apoptosis (Cell Signaling Technology, Massachusetts, USA), anti-neutrophil antibody (Abcam Japan, Tokyo, Japan) to detect neutrophils, and anti-mouse Mac-3 antibody (BD Pharmingen, Tokyo, Japan) to detect macrophages. The procedure for unmasking antigens was antigen dependent, as detailed below.

### 2.7. Immunohistochemical Staining

The concentration of primary antibody in HMGB1, Cleaved Caspase-3, neutrophil, and Mac-3 is 1 : 100, and staining for anti-HMGB1 antibody and Cleaved Caspase-3 antibody used the Dako Envision+ system-HRP labeled polymer anti-rabbit (ready to use) (Dako North America, California, USA) as secondary antibody, and staining for anti-neutrophil antibody and anti-mouse Mac-3 antibody used polyclonal rabbit anti-rat immunoglobulins/HRP (Dako Denmark A/S, Glostrup, Denmark) at a concentration of 1 : 300 with 0.003% mouse serum (Dako Denmark A/S, Glostrup, Denmark) in phosphate-buffered saline (PBS) as secondary antibody. The washing buffer for anti-HMGB1 antibody and Cleaved Caspase-3 antibody was 0.1% Tween 20 in PBS, that for anti-neutrophil antibody was 0.3% TritonX-100 in PBS, and that for anti-mouse Mac-3 antibody was PBS. The applied process was the same for all immunohistochemical staining. In accordance with a previous study [18], after deparaffinization and rehydration, antigen unmasking was accomplished by heating slides in a water bath followed by incubation in sodium citrate buffer (10 mM sodium citrate, 0.05% Tween 20, pH 6.0) for 20 minutes at approximately 100°C. Slides were washed with each washing buffer. Then, slides were incubated with each antibody at 4°C overnight. Slides were then again washed with each washing buffer. For the detection of primary antibodies, slides were incubated with secondary antibody with HRP for 30 minutes at room temperature. Slides were again washed with each washing buffer and then incubated in Dako Liquid DAB+ Substrate Chromogen System (Dako North America, California, USA) (brown chromogen) for 5 minutes or until staining was detected at room temperature. Light hematoxylin counterstaining was applied for 1 minute for the visualization of cell nuclei. Finally, the slides were rinsed in distilled water, dehydrated, cleared, and mounted for analysis. Negative control slides were obtained by omitting each primary antibody.

### 2.8. Microscopic Observations

**Figure 2:**
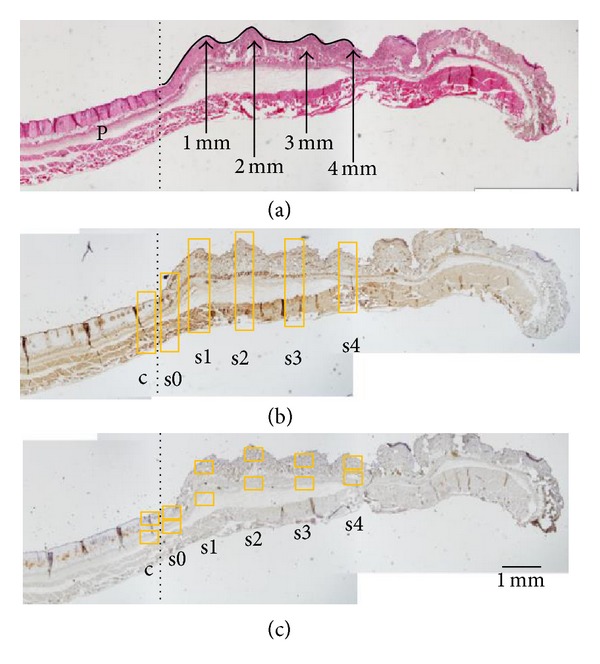
This figure shows the location of histological observations on the right sides of wounds. The zone of coagulation is located on the left side of the dashed line. On the slide of H&E staining (a), the zone of stasis is located up to 2 mm from the end of the zone of coagulation, the zone of hyperemia is located up to 3 mm from the end of the zone of coagulation, and normal skin structures are observed in the zone 4 mm outside the end of the zone of coagulation. P: panniculus carnosus muscle. On the slide of immunohistological staining for necrosis (b), we named five locations: c, the end of the zone of coagulation; s0, the start of the zone of stasis; s1, the site 1 mm from the border between the end of the zone coagulation and the start of the zone of stasis; s2, the location 2 mm from the border between the end of the zone of coagulation and the start of the zone of stasis; s3, the location 3 mm from the border between the end of the zone of coagulation and the start of the zone of stasis; and s4, the location 4 mm from the end of the zone of coagulation (the location near normal skin). We calculated the proportions of necrotic and apoptotic cells in c–s4 for each cell/tissue type (keratinocytes, appendages, cells in dermis, vascular endothelial cells, and adipocytes). On the slide of immunohistochemical staining for neutrophils (c), we identified the 5 locations, c–s4, and counted the numbers of neutrophils and macrophages in these locations per layer (dermis, hypodermis). Additionally, we calculated the overall proportion or number for the immunostaining by summing the proportions or numbers per cell/tissue type or layer for each location and calculating the averages.

Firstly, we observed the right side of each wound by H&E staining and defined the three zones described by Jackson [2] by comparison to previous studies (Figure 2(a)) [20–23]. From this observation, we judged the zone of stasis to be located up to 2 mm from the end of the zone of coagulation and the zone of hyperemia to be located up to 3 mm from the end of the zone of coagulation, and, in the zone 4 mm or more from the end of the zone of coagulation, normal-like skin structures were observed. Therefore, we decided to set 6 points, from c to s4, on each slide with immunohistochemical staining (Figures 2(b) and 2(c)). We defined s4 as the location near normal skin.

We calculated the proportions of necrotic and apoptotic cells in c, s0, s1, s2, s3, and s4 for each cell/tissue type (keratinocytes, appendages, cells in dermis, vascular endothelial cells, and adipocytes) using a light microscope with an object lens at 40x magnification as follows: the proportion of necrotic cells (%) equals total cells minus the number of anti-HMGB1 antibody-positive cells/total cell number; the proportion of apoptotic cells (%) equals the number of Cleaved Caspase-3 antibody-positive cells/total cell number. In addition, we calculated the proportion of necrotic and apoptotic cells at 6 locations of 2 mice (1 mouse, 3 locations) without a burn wound. We defined this as the proportion in normal skin. We counted the numbers of neutrophils and macrophages using a light microscope at 400x magnification and then calculated each number per mm^2^ per layer (dermis, hypodermis). Additionally, we calculated the overall proportion or number in the immunostaining by summing the proportions or numbers per cell/tissue type or layer from each location and calculating the overall averages.

### 2.9. Determination of TNF-**α** in Serum

The TNF-*α* level in mouse serum was measured using Quantikine ELISA (R&D Systems, Minneapolis, USA) according to the manufacturer's protocol. Briefly, 96-well microplates coated with a monoclonal antibody to mouse TNF-**α**were used. Standard wells included 50 *μ*L of serially diluted concentrations of mouse TNF-**α**: 700, 350, 175, 87.5, 43.8, 21.9, 10.9, and 0 pg/mL. Sample wells included 50 *μ*L of serum sample. A total of 50 *μ*L of a buffered protein base was added to all of the wells. The plate was then covered with adhesive film and was incubated for 2 hours at room temperature. All of the wells were washed six times with washing buffer automatically, followed by the addition of 100 *μ*L of a polyclonal antibody against mouse TNF-**α** conjugated to horseradish peroxidase, and the plate was covered with adhesive film and was incubated for 2 hours at room temperature. All of the wells were washed six times with a washing buffer automatically; finally, 100 *μ*L of substrate solution was added to all of the wells, and the plate was incubated for 30 minutes at room temperature. The enzyme reaction was stopped quickly by pipetting 100 *μ*L of stop solution into each well, and the plate was then read immediately using a microplate reader (SUNRISE-BASIC TECAN, TECAN Austria GmbH, Grödig, Austria) at 450 nm and 550 nm.

### 2.10. Statistical Analysis

Data are expressed as mean ± SD and were analyzed using JMP 8.0.1 (SAS, USA) (ANOVA, multiple comparison Tukey-Kramer). Differences were considered significant at *P* < 0.05.

## 3. Results

### 3.1. Macroscopic Observations of Burn Wounds

When we changed each dressing every day, honey and SSD remained on the wound surfaces, the wounds were moist, and gauzes with honey and SSD covering the wounds were easily removed.

**Figure 3:**
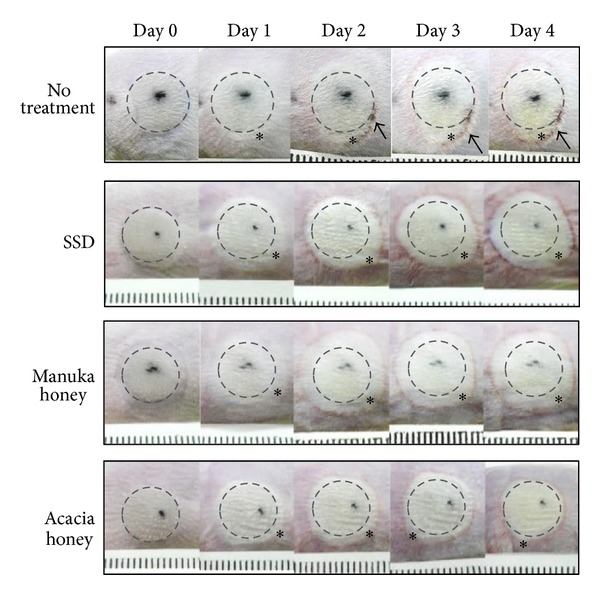
This figure shows the process of wound progression on macroscopic observation. Dotted circles indicate the bottom of the weight used for wounding. Note the zone of stasis (*) expanding over time in all groups. Arrows show scar. Signs of infection were not observed in any wounds. The rulers indicate gradations of 1 millimeter.

On day 0, the burned area turned white in color, and this area was almost the same size as the weight used for wounding (Figure 3). This area corresponds to the zone of coagulation because it has suffered direct heat damage. On day 1, the wounds increased in area in all groups. The area of expansion on day 1 revealed a bright white color compared with the zone of coagulation. This zone corresponds to the zone of stasis because it turned white and fell into necrosis. On day 2, the areas of the wounds continued to increase in all groups, and the new area of expansion revealed a bright white color compared with the zone of coagulation. Some wound edges revealed redness, and, on day 3, complete red rings formed around the wound edges in all groups. This zone corresponds to the zone of hyperemia because this redness reveals increased blood flow by patent blood vessels. On day 4, all wounds stopped increasing in area. All groups revealed almost the same characteristics; however, the SSD group revealed intense redness around the wound edges, and the no treatment group formed a scar around the wound edges.

### 3.2. The Expanded Wound Areas

**Figure 4:**
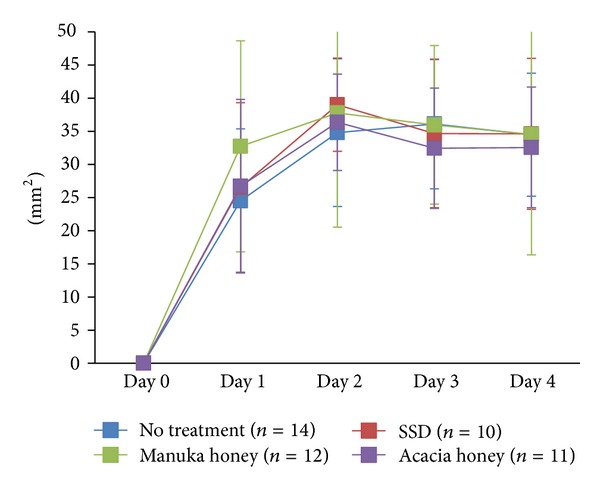
This figure shows the enlarged wound area in each group. Although the size in the acacia honey group seems to be smaller than that in the other groups on days 3 and 4, there were no significant differences between all groups.

In all groups, wound areas rapidly increased; they peaked on day 2 in the SSD, manuka honey, and acacia honey groups and on day 3 in the no treatment group (Figure 4). Although wound areas in the acacia honey group seemed to be smaller than those in the other groups, there were no significant differences between them.

### 3.3. The Characteristics of Each Zone of Burn Wound by H&E Staining

Table 1 shows the characteristics of each zone of burn wounds by H&E staining. From our observations, the location of c corresponded to the zone of stasis, the locations of s0, s1, and s2 corresponded to the zone of stasis, the location of s3 corresponded to the zone of hyperemia, and the location of s4 showed almost normal skin characteristics. The reasons why we judged each location in this way are detailed below.

**Figure 5:**
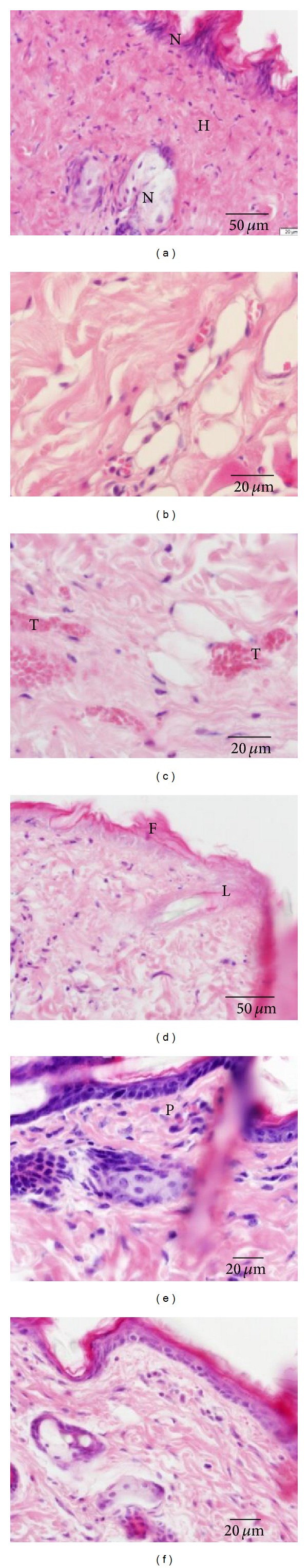
In the location of c (see Figure 1), the epidermis, appendages, and cells in dermis show necrosis (a). In the location of s0, normal blood vessels can be observed (b). On day 4, blood vessels reveal thrombosis at the location of s0 (c). Epidermis and appendage lose their nuclei and the wound surface is covered with eosinophilic film-like material in the location of s3 (d). Patent blood vessels in the superficial dermis are observed on day 3 in the location of s3 (e). In the location of s4, most cells of each type retain normal structures during observation (f). N: necrosis (pyknosis, elongated nuclei, and swelling), H: hyalinized collagen, T: thrombosis, L: loss of nuclei, F: eosinophilic film-like material, and P: patent blood vessel.

 In the location of c, most cells of each type showed necrosis (Figure 5(a)), which was the same as in previous studies [20–23]. We could not observe patent blood vessels in this area, as reported by Jackson [2]. In the locations of s0, s1, and s2, some or most cells of each type seemed to have normal structures at an early stage. We could observe blood vessels, some of which revealed coagulation, bleeding, or thrombosis at an early stage (Figure 5(b)), while the others were normal. On day 4, blood vessels revealed thrombosis or bleeding (Figure 5(c)), and other cell/tissue types revealed necrosis, especially in the epidermis and appendage, in which cells lost their nuclei, and wound surfaces were covered with an eosinophilic film-like material (Figure 5(d)). Only collagen in the dermis kept its individual fibers. In these areas, the blood flow was maintained at an early stage; however, cell necrosis spatiotemporally progressed until day 3 or 4, as also described by Jackson [2]. In the location of s3, most cells of each type seemed to have normal structures. Notably, we observed bleeding or patent blood vessels in the superficial dermis on day 3 (Figure 5(e)). We think that these characteristics correspond to the red ring around the edges or the redness in the zone of hyperemia, as described by Jackson [2]. In the location of s4, most cells of each type retained a normal structure during observation (Figure 5(f)). All groups showed the same characteristics during observations.

### 3.4. The Proportions of Necrotic Cells in Each Zone of Burn Wound

**Figure 6:**
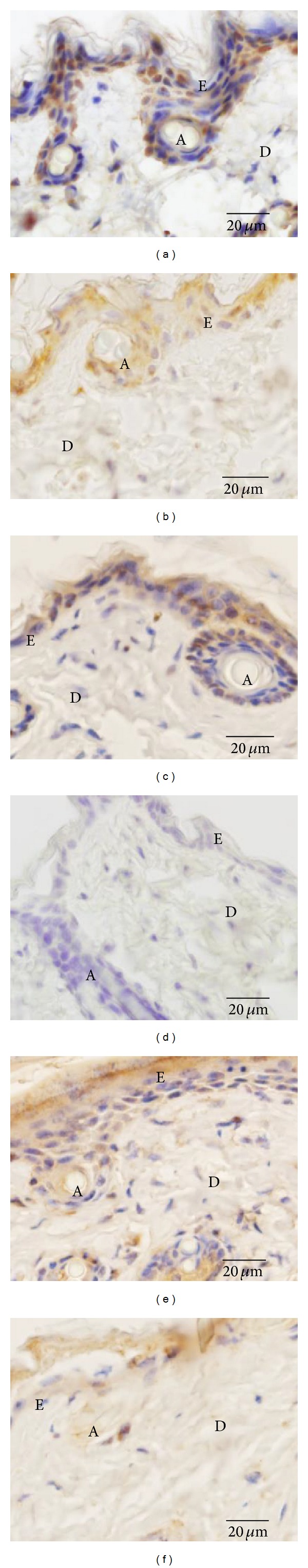
Brown nuclei (positive for anti-HMGB1 antibody) indicate normal cells, while blue nuclei (negative for anti-HMGB1 antibody) reveal necrotic cells. Normal skin (a) has a lot more normal nuclei than all other groups. On day 1, the acacia honey group (c) has a lot more normal nuclei than the SSD group (b) in the location of s1 (see Figure 1). On day 4, the no treatment (e) and manuka honey (f) groups have a lot of necrotic nuclei in the location of s1. Negative control slide (d) is not stained. E: epidermis, A: appendage, and D: dermis.

Table 2 shows all of the results regarding the proportions of necrotic cells. Necrotic cells stained with anti-HMGB1 antibody were observed for all cell/tissue types at all locations (Figure 6). In the location of c corresponding to the zone of coagulation, the proportions of necrotic cells were about 90–100%; that is, most cells revealed necrosis. These results were the same as the observation by H&E staining. In the locations of s0–s2 corresponding to the zone of stasis, the proportions of necrotic cells were about 80–90%. These results were also the same as the observation by H&E staining. In the location of s3 or s4 corresponding to the zone of hyperemia or the location near normal skin, the proportions of necrotic cells were about 70–90%, which differed from the observation by H&E staining. These areas revealed almost normal characteristics by H&E staining; however, the proportions of necrotic cells were actually almost the same as in the zone of stasis. The proportions of necrotic cells in all zones were higher than those in normal skin (*P* values not shown).

At 30 minutes after wounding, the proportions of necrotic cells in the manuka honey and acacia honey groups were significantly lower than those in the no treatment and SSD groups, although significant differences were not identified for all cell/tissue types and locations. The overall proportions of necrotic cells for all cell/tissue types and locations in the manuka honey and acacia honey groups were significantly lower than those in the no treatment and SSD groups (manuka honey versus no treatment or SSD: *P* = 0.0018, <0.0001; acacia honey versus no treatment or SSD: *P* = 0.0119, <0.0001, resp.). On day 1, the proportions of necrotic cells in the manuka honey and acacia honey groups were significantly lower than those in the SSD group, although there were no significant differences for all parameters and locations. The overall proportions of necrotic cells for all parameters and locations in the manuka honey and acacia honey groups were significantly lower than those in the SSD group (manuka honey versus SSD: *P* = 0.0029; acacia honey versus SSD: *P* = 0.0008). On day 2, the proportions of necrotic cells in the manuka honey and acacia honey groups were significantly lower than those in the SSD group, although there were no significant differences for all cell/tissue types and locations. The overall proportions of necrotic cells for all cell/tissue types and locations in the manuka honey and acacia honey groups were significantly lower than those in the SSD group (manuka honey versus SSD: *P* = 0.0035; acacia honey versus SSD: *P* = 0.0003). On day 3, the proportions of necrotic cells in the manuka honey and acacia honey groups were significantly lower than those in the SSD group, although there were no significant differences for all cell/tissue types and locations. There were no significant differences between the manuka honey and no treatment groups and between the acacia honey and no treatment groups; however, the overall proportions of necrotic cells for all cell/tissue types and locations in the manuka honey and acacia honey groups were significantly lower than those in the no treatment and SSD groups (manuka honey versus no treatment or SSD: *P* = 0.0271, 0.0137; acacia honey versus no treatment or SSD: *P* = 0.0677, 0.0364, resp.). On day 4, the proportions of necrotic cells in all groups did not show clear significant differences, and also the overall proportions of necrotic cells in all cell/tissue types and locations in all groups were almost the same and there were no significant differences between all groups.

### 3.5. The Proportions of Apoptotic Cells in Each Zone of Burn Wound

Table 3 shows all of the results regarding the proportions of apoptotic cells. Apoptotic cells stained with Cleaved Caspase-3 antibody were observed much more rarely than necrotic cells. The proportions of apoptotic cells in each zone were not higher than those in normal skin. At 30 minutes and 1, 3, and 4 days after wounding, the proportions of apoptotic cells in some groups showed significant differences; however, the overall proportion of apoptotic cells for all cell/tissue types and locations in all groups did not show significant differences. Only on day 3 was the overall proportion of apoptotic cells for all parameters and locations in the no treatment group higher than that in the SSD group (*P* = 0.0070).

### 3.6. The Number of Neutrophils in Each Zone of Burn Wound

**Figure 7:**
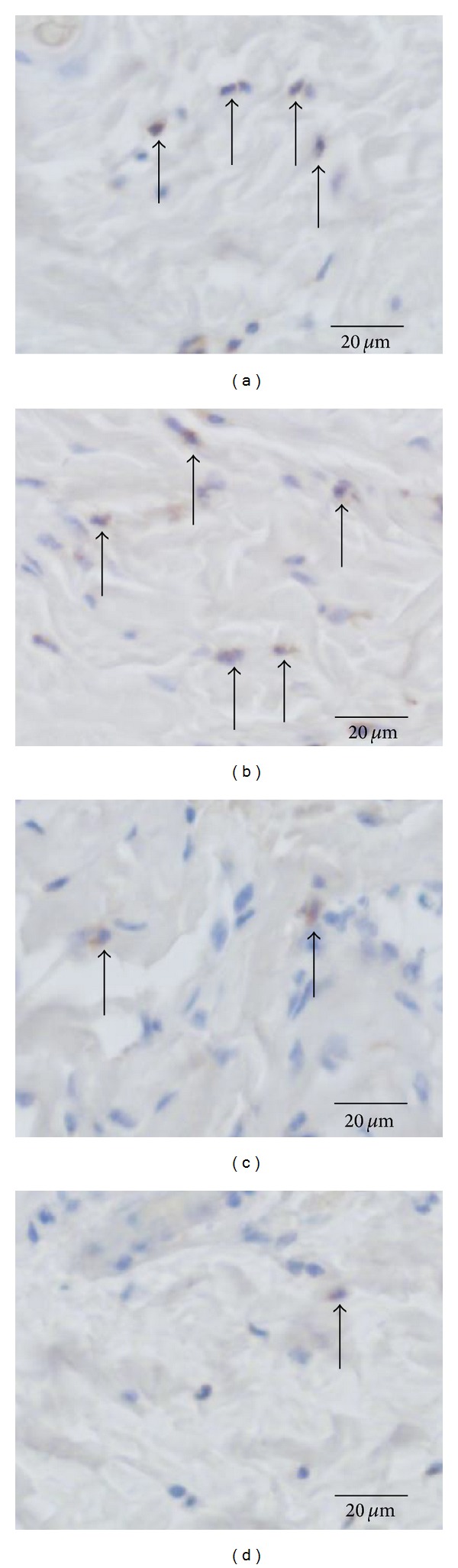
Arrows reveal neutrophils. In the location of s1 (see Figure 1), the numbers of neutrophils in manuka honey (c) and acacia honey groups (d) in the dermis on day 2 are decreased compared with those in the no treatment (a) and SSD groups (b) on histological observation.

The left column in Table 4 shows all of the results regarding the number of neutrophils. At 30 minutes after wounding, the numbers of neutrophils showed significant differences between some groups. However, the overall number of neutrophils in all layers and locations in all groups did not show significant differences. On day 1, the number of neutrophils in the manuka honey group in particular increased in the locations of s0–s2 corresponding to the zone of stasis, and the number of neutrophils in the no treatment group increased in the locations of s3 and s4 corresponding to the zone of hyperemia and the location near normal skin. The overall number of neutrophils in the acacia honey group was significantly smaller than that in the no treatment and manuka honey groups (*P* = 0.0692, 0.0018, resp.). On day 2, the numbers of neutrophils in the manuka honey and acacia honey groups seemed to be smaller than those in the no treatment and SSD groups (Figure 7), and there were some significant differences between the SSD and manuka honey groups and between the SSD and acacia honey groups; in addition, the overall numbers of neutrophils in all layers and locations in the manuka honey and acacia honey groups were smaller than those in the SSD group (*P* = 0.0386, 0.0045, resp.). On day 3, the number of neutrophils in the no treatment group seemed to increase in the location of c corresponding to the zone of coagulation in the dermis, and the number of neutrophils in the SSD group also increased in the location of s2 corresponding to the zone of stasis in the dermis. However, the overall number of neutrophils in all layers and locations in the acacia honey group tended to be smaller than that in the no treatment group (*P* = 0.0965). On day 4, there were no significant differences between all groups in the number of neutrophils, and also the overall number of neutrophils in all layers and locations in all groups did not show significant differences.

### 3.7. The Number of Macrophages in Each Zone of Burn Wound

The right column in Table 4 shows all of the results regarding the number of macrophages. At 30 minutes after wounding, there were some significant differences between the no treatment and SSD groups, between the no treatment and manuka honey groups, and between the no treatment and acacia honey groups; in addition, in terms of the overall numbers of macrophages in all layers and locations, those in the SSD, manuka honey and acacia honey groups were smaller than those in the no treatment group (*P* = 0.0519, 0.0044, and 0.0156, resp.). On days 1, 2, and 3, there were some significant differences in the numbers of macrophages, but the overall numbers of macrophages in all layers and locations in all groups did not show significant differences. On day 4, the numbers of macrophages in the manuka and acacia honey groups were smaller than those in the no treatment group in the locations of c–s1 corresponding to the zone of coagulation and stasis, and the numbers of macrophages in the no treatment, manuka honey, and acacia honey groups were smaller than those in the SSD group at the locations of s2 and s3 corresponding to the zones of stasis and hyperemia. The overall numbers of macrophages in all layers and locations in the manuka honey and acacia honey groups tended to be smaller than those in the no treatment group (*P* = 0.0844, 0.0871, resp.).

### 3.8. The Level of Serum TNF-**α** in All Groups

Table 5 shows the level of serum TNF-**α** in each group. Although this level in the manuka honey group tended to be higher than that in the acacia honey (*P* = 0.0739) and no treatment groups (*P* = 0.0646) on day 2 or 4, there were no clear significant differences between all groups.

## 4. Discussion

### 4.1. The Effects of Honey on Burn Wound Progression

A burst of free radicals in burn trauma exacerbates xanthine oxidase activity, producing an overwhelming tissue damage like lipid peroxidation, protein oxidation, and oxidative DNA damage [11, 13, 14], which further promotes necrosis in the zone of stasis and causes the area of burn wound to enlarge. On the other hand, in burn wounds, honey has been revealed to decrease wound area, to have an antibacterial effect [4], and to promote reepithelialization [5] compared with hydrofiber silver or silver sulfadiazine (SSD); honey also shortens the inflammatory phase. Moreover, Molan [10] estimated that the anti-inflammatory action of honey reduces the damage caused by the free radicals that arise from inflammation and thus prevents further necrosis. Therefore, we tried to clarify the effects of honey on burn wounds in the inflammatory phase. Thus, in the present study, we counted the numbers of neutrophils and macrophages around the zone of coagulation to estimate the free radical damage and also measured the serum TNF-**α** to evaluate systemic inflammation. Contrary to our hypothesis, in the manuka honey and acacia honey groups, burn wound progression was not prevented because all wounds increased in area after wounding; there were no significant differences in the expanded wound areas between all groups, and the necrotic and apoptotic cells in the manuka honey and acacia honey groups increased in number around the zone of coagulation as well as in the no treatment and SSD (antibacterial agent) groups. However, some types of honey have been shown to be effective intreating burn wounds [4, 5], and it has been said that, as honey is a natural product, its characteristics associated with wound healing may be affected by the species of bee, geographical location, and botanical origin, as well as processing and storage conditions [24]. Therefore, we think that other types of honey are needed to research the effects on burn wound progression. 

Inflammation is characterized by redness, swelling, heat, and pain [11]. In the present study, in the no treatment group, a scar was formed on the wound edge, while the SSD group exhibited intense redness at the wound edge compared with the other groups; however, the manuka honey and acacia honey groups did not exhibit scarring and showed weak redness. Gates and Holloway [25] reported that incision wounds treated with moist wound environment dressings were associated with fewer complaints of pain. Moreover, Biglari et al. [26] reported that Medihoney produced from manuka honey significantly reduced perceived pain levels in a multicenter prospective observational study, and Molan [10] also stated that honey reduced pain in burn wounds in her review. Therefore, the manuka and Japanese acacia honey that we used may decrease the pain from inflammation in burn wounds.

### 4.2. The Characteristics of Each Zone of Burn Wound Compared with Jackson's Observations [2]

Jackson focused on flame and scald burns in humans, while our study dealt with scald burns in mice. Although the types of burn differ between Jackson's work and our own, it is important to describe the detailed characteristics of each zone of a burn wound by comparison to Jackson's observations because there have been few articles on this issue.

 We show the characteristics of each zone of a burn wound in Table 6. The zone of coagulation was white in color macroscopically and showed coagulated necrosis histologically in both Jackson's work and our own. Jackson described that the zone of stasis turned from red to white color, while; in our study, the zone of stasis rapidly turned white without a change to red on day 1. Jackson described that whiteness on the skin resulted from a loss of blood vessels, and we observed thrombosis and high proportions of necrotic cells in the zone of stasis; we therefore think that burn wounds in mice may turn white more rapidly than those in humans because of the rapid loss of oxyhemoglobin in this zone. In addition, the characteristics of the zone of stasis in our study included loss of nuclei in the epidermis and appendages and the covering of the wound surface with an eosinophilic film-like material. Although Jackson did not describe the characteristics of the epidermis, some studies described the detachment of the epidermis in animal burn models [15, 16]. Therefore, the epidermis in the zone of stasis in animal models is detached or lost. In the zone of hyperemia, Jackson described the loss of epidermis, while we could observe normal epidermis. We think that these differences result from how the burn wounds were formed. Thermal intensity was not constant in the humans in the previous study, while, in the animals in this study, we could control the heated area and clearly distinguish it from the unheated area. We estimate that the zone of hyperemia in Jackson's work suffered a little thermal damage, which contributed to the loss of epidermis. In contrast, the zone of hyperemia in our animal model retained the epidermis. Additionally, species differences may also have contributed to the observed differences. 

 Moreover, we observed redness around the wound edges and normal skin structures without necrosis, like pyknosis or elongated nuclei, as well as patent blood vessels in the zone of hyperemia. These findings were the same as those by Jackson. However, the proportions of necrotic cells in the location of s3 corresponding to the zone of hyperemia were about 80–90%, while those in normal skin were about 50–60%. In the location of s3, the loss of nuclei was not observed, and, considering the proportions of necrotic cells in normal skin, about 30% of all cells in this zone fell into necrosis. In addition, about 30% of all cells in the location of s4 did so. Therefore, it is revealed that the zone of hyperemia and its surroundings in a burn wound fall into necrosis, which contributes to burn wound progression, although these areas seem to retain normal structures on macroscopic or H&E staining observations. Although this finding may only be observed in an animal burn model, it suggests that necrosis in the zone of hyperemia and its surroundings can be detected by immunostaining, so we must consider these differences if we apply the findings from animal models to a clinical setting. In a clinical setting, the correct diagnosis often depends on the surgeon's experience [1]. Considering our results, the zone of hyperemia and its surroundings may also fall into necrosis in a clinical setting, so we need to discuss whether debridement of these tissues can promote wound healing.

In this study, the limitations include the use of only two types of honey, although honey includes many types globally, and the fact that the research involved using mice with only deep burn wounds. Different conditions, different types of honey, and different animals or depths of burn wounds will be needed to evaluate more comprehensively the effects of honey on burn wounds.

## 5. Conclusions

Treatment of deep burn wounds with manuka honey and Japanese acacia honey cannot prevent burn wound progression. Comparing Jackson's observations with our own, it seems that the differences between humans and animals in terms of the different zones of burn wounds may be due to the differences in how the wound was formed or due to species differences; we need to consider these differences if we apply the findings from animal models to a clinical setting. It is revealed that the zone of hyperemia and its surroundings in burn wounds fall into necrosis, which contributes to burn wound progression, although these areas seem to retain normal structures from macroscopic or H&E staining observations.

## Figures and Tables

**Table 1:** The characteristics of each zone on burn wound with H&E staining.

	30 min	Day 1	Day 2	Day 3	Day 4
c					
Epidermis	N +++	N +++	N +++	N +++	N +++
Appendage	N +++	N +++	N +++	N +++	N +++
Dermis	H +++	H +++	H +++	H +++	H +++
Blood vessel	D ++	D +++	D +++	D +++	D +++
Adipocyte	D ++	D +++	D +++	D +++	D +++

s0					
Epidermis	N ++	N ++	N ++	L ++	L +++
Appendage	N ++	N ++	N ++	L ++	L +++
Dermis	H +	H +	H +	H +	H +
Blood vessel	C +/−	CB +	C +	CT +++	CT +++
Adipocyte	D ++	D +++	D +++	D +++	D +++

s1					
Epidermis	N +	N +	L +	L ++	L +++
Appendage	N +	N +	L +	L ++	L +++
Dermis	H −	H −	H −	H −	H −
Blood vessel	C +/−	CB +	T +	T ++	T ++
Adipocyte	D ++	D +++	D +++	D +++	D +++

s2					
Epidermis	N −	N −	L +	L +	L ++
Appendage	N −	N −	L +	L +	L ++
Dermis	H −	H −	H −	H −	H −
Blood vessel	T +/−	T +/−	BT +	BT +	BT +
Adipocyte	D +	D +	D ++	D ++	D

s3					
Epidermis	N −	N −	N −	N −	N −
Appendage	N −	N −	N −	N −	N −
Dermis	H −	H −	H −	H −	H −
Blood vessel	T +/−	T +/−	T +/−	PB ++	B ++++
Adipocyte	D +	D +	D +	D ++	D ++

s4					
Epidermis	N −	N −	N −	N −	N −
Appendage	N −	N −	N −	N −	N −
Dermis	H −	H −	H −	H −	H −
Blood vessel	T +/−	T +/−	T +/−	T +/−	T +/−
Adipocyte	D +	D +	D +	D +	D ++

N: necrosis (pyknosis, elongated nucleus, and swelling), L: loss of nucleus, D: destruction of structure, H: hyalinized collagen, B: bleeding, C: coagulation, T: thrombus, and P: patent blood vessel. −: negative, +/−: very mild, +: mild, ++: moderate, and +++: severe.

**Table 2:** The proportions of necrotic cells in each group.


Group	Epidermis (*n*)	30 min	Day 1	Day 2	Day 3	Day 4	Appendage (*n*)	30 min	Day 1	Day 2	Day 3	Day 4

c												
No treatment	5–10	96.5 ± 7.4	98.4 ± 3.6	100.0 ± 0.0	100.0 ± 0.0	100.0 ± 0.0	5–9	100.0 ± 0.0	100.0 ± 0.0	100.0 ± 0.0	100.0 ± 0.0	100.0 ± 0.0
SSD	8–11	100.0 ± 0.0	100.0 ± 0.0	100.0 ± 0.0	100.0 ± 0.0	100.0 ± 0.0	7–10	100.0 ± 0.0	100.0 ± 0.0	100.0 ± 0.0	100.0 ± 0.0	100.0 ± 0.0
Manuka honey	8–12	99.7 ± 1.0	100.0 ± 0.0	100.0 ± 0.0	100.0 ± 0.0	100.0 ± 0.0	8–11	100.0 ± 0.0	100.0 ± 0.0	100.0 ± 0.0	100.0 ± 0.0	100.0 ± 0.0
Acacia honey	9–11	97.1 ± 4.8	100.0 ± 0.0	100.0 ± 0.0	100.0 ± 0.0	100.0 ± 0.0	8–11	100.0 ± 0.0	100.0 ± 0.0	100.0 ± 0.0	100.0 ± 0.0	100.0 ± 0.0
s0												
No treatment	5–10	96.1 ± 7.9	94.1 ± 10.6	98.3 ± 3.8	99.6 ± 1.1	97.8 ± 2.8	5–9	93.4 ± 8.6	91.4 ± 12.8	93.4 ± 9.6	99.0 ± 2.7	96.8 ± 5.4
SSD	8–11	98.2 ± 4.2	99.7 ± 1.0	100.0 ± 0.0	100.0 ± 0.0	100.0 ± 0.0	7–10	91.5 ± 11.8^cde′′^	97.4 ± 6.4	100.0 ± 0.0	100.0 ± 0.0	100.0 ± 0.0
Manuka honey	8–12	98.8 ± 3.0	100.0 ± 0.0	100.0 ± 0.0	99.0 ± 3.4	99.5 ± 1.4	8–11	93.3 ± 11.0	92.5 ± 6.0	94.8 ± 9.6	98.9 ± 3.8	100.0 ± 0.0
Acacia honey	9–11	87.7 ± 5.5^*β*′^	93.6 ± 10.2	97.5 ± 4.4	97.6 ± 5.4	97.7 ± 4.0	8–11	82.7 ± 10.8 ^c′de′^	89.0 ± 7.0^*β*′′^	96.5 ± 8.0	94.6 ± 8.6^*β*′′^	97.5 ± 4.8
s1												
No treatment	5–10	83.9 ± 11.0	88.4 ± 11.9	80.9 ± 4.0^*β*′^	93.5 ± 7.9	92.4 ± 5.4	5–9	85.8 ± 6.6	84.4 ± 9.2	86.7 ± 10.6	87.9 ± 9.6	83.2 ± 10.8
SSD	8–11	95.5 ± 5.2	94.3 ± 4.7	96.5 ± 4.6	96.0 ± 6.1	92.9 ± 8.2	7–10	90.0 ± 5.0	91.4 ± 7.7	94.3 ± 8.4	93.2 ± 10.8	93.7 ± 6.9
Manuka honey	8–12	81.2 ± 8.7^*β*′^	94.8 ± 3.1	86.8 ± 12.1^*β*^	93.1 ± 5.4	94.5 ± 7.5	8–11	78.8 ± 9.9	86.0 ± 3.8	82.0 ± 10.9^*β*^	84.1 ± 17.9	89.2 ± 15.3
Acacia honey	9–11	8.9 ± 10.9^*β*′^	82.4 ± 8.2 ^*β*′*γ*′^	85.5 ± 4.5^*β*′^	92.7 ± 5.6	91.0 ± 7.6	8–11	85.7 ± 5.1	84.7 ± 9.6	80.4 ± 8.0^*β*^	88.8 ± 12.6	88.6 ± 12.4
s2												
No treatment	5–10	83.2 ± 9.4	82.8 ± 12.2^*β*′′^	82.6 ± 7.6^*β*^	87.9 ± 6.6	86.0 ± 5.5	5–9	84.9 ± 5.4	84.5 ± 4.9	80.4 ± 9.1	81.5 ± 9.2	76.5 ± 17.1
SSD	8–11	94.6 ± 3.3	92.9 ± 5.1	93.4 ± 4.3	92.3 ± 5.2	91.6 ± 6.5	7–10	84.4 ± 8.0	84.7 ± 9.8	86.5 ± 8.5	83.5 ± 9.2	83.7 ± 15.5
Manuka honey	8–12	78.1 ± 10.2^*β*′^	88.9 ± 9.8	87.3 ± 6.3	87.3 ± 7.0	89.3 ± 5.7	8–11	75.5 ± 11.7	81.0 ± 5.0	77.2 ± 11.5	73.2 ± 13.9	78.3 ± 7.3
Acacia honey	9–11	75.1 ± 11.2^*β*′^	76.1 ± 8.0^*β*′*γ*′^	82.7 ± 7.7^*β*′^	87.1 ± 7.1	83.7 ± 4.4^*β*^	8–11	82.4 ± 3.1	78.4 ± 12.7	79.6 ± 8.4	79.9 ± 14.5	83.8 ± 9.8
s3												
No treatment	5–10	78.3 ± 8.6	83.0 ± 9.1^*β*′′^	87.0 ± 8.9	87.3 ± 5.1	86.2 ± 7.4	5–9	80.6 ± 9.4	79.5 ± 8.0	81.3 ± 6.2	78.9 ± 8.9	80.2 ± 10.9
SSD	8–11	91.9 ± 4.4	92.5 ± 5.7	89.7 ± 6.2	88.7 ± 6.0	87.2 ± 7.7	7–10	87.6 ± 5.2	87.9 ± 6.3	81.4 ± 10.8	82.0 ± 12.3	81.7 ± 7.1
Manuka honey	8–12	76.0 ± 13.7^*β*′^	83.3 ± 8.8^*β*^	86.0 ± 5.1	87.3 ± 8.6	86.3 ± 4.9	8–11	72.3 ± 11.1 ^*β*′*θ*′′^	72.3 ± 10.8^*β*′*θ*^	72.0 ± 12.2	78.4 ± 9.8	78.1 ± 5.4
Acacia honey	9–11	75.2 ± 10.1^*β*′^	79.0 ± 6.9^*β*′^	79.0 ± 7.8^*β*′^	83.0 ± 4.5	87.1 ± 3.4	8–11	83.0 ± 7.4	82.3 ± 6.9	72.3 ± 8.8^ab^	78.0 ± 7.7	80.1 ± 7.1
s4												
No treatment	5–10	79.4 ± 6.0	88.1 ± 7.0	90.4 ± 4.2	86.7 ± 8.1	88.6 ± 7.0	5–9	88.4 ± 6.1	80.4 ± 8.7	86.2 ± 8.7	77.9 ± 10.5	79.4 ± 8.3
SSD	8–11	90.6 ± 5.4	88.5 ± 7.2	90.5 ± 6.6	92.2 ± 4.4	89.5 ± 3.2	7–10	86.6 ± 8.6	86.5 ± 5.2	81.7 ± 11.5	85.3 ± 12.9	83.7 ± 9.8
Manuka honey	8–12	77.5 ± 10.1^*β*′^	84.0 ± 6.9	84.6 ± 5.3	89.7 ± 8.7	88.0 ± 6.0	8–11	74.8 ± 11.7^*α*^	78.2 ± 7.9	73.9 ± 14.1	76.1 ± 10.6	83.3 ± 8.2
Acacia honey	9–11	82.8 ± 6.9	83.9 ± 8.1	80.0 ± 8.5^*αβ*′^	82.7 ± 9.3^*β*^	84.2 ± 8.1	8–11	80.5 ± 10.7	78.5 ± 9.4	79.0 ± 10.7	82.4 ± 8.6	83.5 ± 6.9

Group	Dermis (*n*)	30 min	Day 1	Day 2	Day 3	Day 4	Endothelial cell (*n*)	30 min	Day 1	Day 2	Day 3	Day 4

c												
No treatment	6–10	99.7 ± 1.0	100.0 ± 0.0	100.0 ± 0.0	100.0 ± 0.0	100.0 ± 0.0	6–9	95.7 ± 8.1	100.0 ± 0.0	100.0 ± 0.0	100.0 ± 0.0	100.0 ± 0.0
SSD	8–11	100.0 ± 0.0	99.8 ± 0.7	100.0 ± 0.0	100.0 ± 0.0	100.0 ± 0.0	7–11	93.3 ± 6.7^de′′^	98.0 ± 6.3	98.3 ± 5.3	100.0 ± 0.0	100.0 ± 0.0
Manuka honey	7–11	99.1 ± 1.4^c′′de′′^	99.8 ± 0.6	100.0 ± 0.0	100.0 ± 0.0	100.0 ± 0.0	6–10	90.7 ± 9.7^cd′e^	98.0 ± 6.3	100.0 ± 0.0	100.0 ± 0.0	100.0 ± 0.0
Acacia honey	9–11	97.2 ± 4.0^*α*′′*β*^	99.9 ± 0.4	99.7 ± 0.9	100.0 ± 0.0	97.9 ± 6.4	5–9	84.4 ± 15.0	93.4 ± 9.8	95.8 ± 7.7	97.5 ± 7.1	96.7 ± 7.5
s0												
No treatment	6–10	97.9 ± 3.0	96.2 ± 5.8	96.0 ± 4.7^*βγ*′′^	100.0 ± 0.0	98.2 ± 2.4	6–9	98.8 ± 3.1	100.0 ± 0.0	97.2 ± 8.3	100.0 ± 0.0	100.0 ± 0.0
SSD	8–11	99.5 ± 1.5	99.3 ± 2.2	100.0 ± 0.0	100.0 ± 0.0	99.3 ± 1.3	6–11	95.4 ± 7.6	96.8 ± 7.8	96.7 ± 8.2	100.0 ± 0.0	100.0 ± 0.0
Manuka honey	7–11	97.6 ± 7.8	96.9 ± 2.6	100.0 ± 0.0	99.1 ± 2.3	99.6 ± 1.2	5–9	88.2 ± 12.2	91.8 ± 10.9	100.0 ± 0.0	98.0 ± 6.1	97.1 ± 6.4
Acacia honey	9–11	95.4 ± 7.2	96.4 ± 3.9	97.0 ± 3.5^*β*′′^	98.5 ± 2.7	93.9 ± 10.6	5–11	88.4 ± 14.5	85.4 ± 9.4 ^*α*′*β*^	88.2 ± 13.4	92.1 ± 13.9	91.8 ± 12.6
s1												
No treatment	6–10	100.0 ± 0.0	95.6 ± 3.2	94.7 ± 4.9	96.2 ± 5.9	92.4 ± 8.8^a^	5–10	98.1 ± 5.0	95.0 ± 11.2	95.2 ± 6.2	100.0 ± 0.0	97.2 ± 6.6
SSD	8–11	99.3 ± 1.5	98.7 ± 2.7	99.7 ± 1.0	97.5 ± 3.8	96.6 ± 4.3	7–10	94.3 ± 9.1	96.5 ± 5.6	94.1 ± 11.6	98.0 ± 6.3	98.0 ± 5.4
Manuka honey	7–11	95.3 ± 4.1^*αβ*^	95.8 ± 3.8	94.3 ± 3.3^*β*′′^	95.9 ± 5.2	94.1 ± 7.0	7–10	84.8 ± 9.4^*α*d′′^	86.8 ± 11.7	86.9 ± 14.4	97.8 ± 3.7	93.9 ± 11.5
Acacia honey	9–11	94.7 ± 4.4^*α*′*β*^	91.4 ± 4.1^*α*′′*β*′*γ*^	91.5 ± 6.3^*β*′^	95.2 ± 5.1	93.7 ± 8.2	6–9	86.5 ± 12.6	86.8 ± 12.5	86.3 ± 12.2	94.0 ± 8.2	92.5 ± 12.3
s2												
No treatment	6–10	99.5 ± 1.2	95.7 ± 5.0	95.6 ± 5.5	97.0 ± 3.3	92.1 ± 8.5^a^	4–9	96.9 ± 6.2	96.4 ± 7.1	98.5 ± 3.7	98.8 ± 3.5	98.3 ± 2.7
SSD	8–11	100.0 ± 0.0	98.2 ± 3.3	97.7 ± 4.1	95.0 ± 3.2^a^	95.6 ± 4.5^a′′^	7–10	94.3 ± 8.9	96.1 ± 8.7	96.6 ± 7.2	100.0 ± 0.0	96.9 ± 8.8
Manuka honey	7–11	90.7 ± 6.9^*α*′*β*′^	91.9 ± 3.6^*β*′^	95.6 ± 5.5	91.8 ± 6.6	89.3 ± 7.3	4–9	82.7 ± 14.8	86.7 ± 11.0	86.7 ± 11.4	95.1 ± 7.9	96.2 ± 9.4
Acacia honey	9–11	93.7 ± 3.6^*αβ*′^	95.6 ± 3.9	94.2 ± 6.6	93.9 ± 6.9	90.2 ± 9.1	5–9	83.4 ± 13.9^*α*′′^	85.4 ± 16.8	89.5 ± 9.9	95.8 ± 9.4	92.2 ± 8.9
s3												
No treatment	5–10	99.2 ± 1.8	95.3 ± 4.0	97.5 ± 2.5	96.9 ± 2.8	94.2 ± 3.8^a′^	5–10	94.4 ± 7.9	95.8 ± 5.8	100.0 ± 0.0	100.0 ± 0.0	97.8 ± 3.5
SSD	8–11	99.7 ± 0.8	99.1 ± 2.2	97.3 ± 3.6	95.6 ± 6.6	96.0 ± 6.4	4–10	96.4 ± 9.4	98.0 ± 6.3	98.6 ± 3.2	97.1 ± 6.4	95.8 ± 8.3
Manuka honey	7–11	90.1 ± 6.2^*α*′*β*′^	88.6 ± 4.4^*αβ*′*θ*^	92.9 ± 5.7	94.2 ± 5.1	89.2 ± 6.8	4–9	85.4 ± 11.8	84.9 ± 11.6^*β*^	83.1 ± 18.1^*α*′′*β*′′^	94.9 ± 8.7	96.4 ± 7.1
Acacia honey	9–11	94.4 ± 4.3^*αβ*^	93.5 ± 5.5^*β*^	91.8 ± 5.2^*β*^	92.9 ± 6.2	94.5 ± 6.2	4–10	83.0 ± 13.8	88.4 ± 12.9	90.3 ± 8.4	96.1 ± 4.8	95.0 ± 10.0
s4												
No treatment	5–10	98.4 ± 2.1	93.2 ± 9.5	97.2 ± 3.3	95.8 ± 4.0	91.0 ± 6.6^a′′^	4–7	94.7 ± 6.2	92.9 ± 12.0	98.1 ± 3.8	98.2 ± 4.7	94.0 ± 10.3
SSD	8–11	99.4 ± 1.9	99.1 ± 1.7	99.2 ± 1.9	97.6 ± 3.7	96.6 ± 5.3	4–8	95.2 ± 12.6	100.0 ± 0.0	98.6 ± 3.9	94.3 ± 8.8	95.2 ± 5.5
Manuka honey	7–11	88.8 ± 9.3^*α*′*β*′^	90.8 ± 11.2^*β*′′^	96.4 ± 3.5	92.4 ± 6.2^*β*′′^	87.2 ± 5.5^*β*^	4–8	85.6 ± 11.6	86.4 ± 9.8^*β*^	90.6 ± 12.0	93.6 ± 8.7	95.1 ± 7.6
Acacia honey	9–11	93.8 ± 5.3	95.8 ± 5.2	93.0 ± 6.2^*β*^	95.4 ± 5.0	93.8 ± 7.4	4–7	82.0 ± 11.9	88.3 ± 13.0	88.2 ± 7.9^*β*′′^	93.3 ± 14.9	90.8 ± 10.7

Group	Adipose (*n*)	30 min	Day 1	Day 2	Day 3	Day 4	Overall (*n*)	30 min	Day 1	Day 2	Day 3	Day 4

C												
No treatment	6–10	92.5 ± 8.1 ^b′c′d′e′^	100.0 ± 0.0	100.0 ± 0.0	100.0 ± 0.0	100.0 ± 0.0	5–11	93.4 ± 2.6	93.1 ± 5.1	93.6 ± 1.8	95.8 ± 1.8	94.0 ± 3.3
SSD	8–11	100.0 ± 0.0	100.0 ± 0.0	100.0 ± 0.0	100.0 ± 0.0	99.5 ± 1.5	8–11	96.2 ± 2.5	96.6 ± 1.4	96.2 ± 1.5	96.4 ± 1.8	95.8 ± 2.9
Manuka honey	9–11	92.4 ± 8.9 ^*β*′′bcd′e′^	98.9 ± 2.7	98.3 ± 3.4	100.0 ± 0.0	100.0 ± 0.0	7–11	87.6 ± 3.3^*α*′*β*′b′′c′′d′e′^	91.0 ± 1.9^*β*′^	91.3 ± 3.9^*β*′^	93.4 ± 2.8^*αβ*^	93.6 ± 1.9
Acacia honey	9–12	96.9 ± 4.1	98.1 ± 3.9	99.7 ± 1.0	98.7 ± 3.3	98.6 ± 3.4	9–12	88.5 ± 3.1^*αβ*′d′e^	90.5 ± 4.5^*β*′d′′^	90.6 ± 2.9^*β*′^	93.8 ± 1.8^*α*′′*β*^	92.9 ± 1.3
s0												
No treatment	6–10	99.4 ± 1.7	97.4 ± 4.4^d′′e′′^	100.0 ± 0.0	100.0 ± 0.0	100.0 ± 0.0	Normal skin (*n*)					
SSD	8–11	99.5 ± 1.5	100.0 ± 0.0	100.0 ± 0.0	100.0 ± 0.0	99.1 ± 2.5	6	Epidermis	50.2 ± 6.5			
Manuka honey	9–11	91.7 ± 9.9 ^*αβ*c′′de^	96.8 ± 5.6	98.7 ± 2.8	100.0 ± 0.0	100.0 ± 0.0	6	Appendage	47.0 ± 6.6			
Acacia honey	9–12	95.9 ± 5.9	95.7 ± 8.5	99.1 ± 3.0	100.0 ± 0.0	95.9 ± 6.5^*α*′′*γ*′′^	6	Dermis	62.8 ± 6.8			
s1												
No treatment	6–10	99.0 ± 2.1	92.3 ± 12.4	96.9 ± 4.9	99.8 ± 0.7	98.3 ± 5.3	6	Blood vessel	56.8 ± 10.7			
SSD	8–11	97.8 ± 3.7	100.0 ± 0.0	99.5 ± 1.6	99.6 ± 6.1	98.0 ± 4.0	6	Adipose	57.2 ± 8.6			
Manuka honey	9–11	93.7 ± 9.0	95.6 ± 7.1	93.1 ± 7.7^*β*′′^	98.8 ± 3.8	97.3 ± 6.3	6	All	54.8 ± 1.8			
Acacia honey	9–12	93.0 ± 6.5	95.3 ± 6.0	93.8 ± 5.5^*β*′′^	95.6 ± 5.9^*α*′′*β*′′^	96.9 ± 5.6						
s2												
No treatment	5–10	98.6 ± 2.9	91.4 ± 13.4	97.4 ± 5.8	99.1 ± 2.9	97.7 ± 5.1						
SSD	8–11	99.6 ± 1.3	99.6 ± 1.3	98.0 ± 4.4	98.7 ± 5.2	100.0 ± 0.0						
Manuka honey	9–11	86.1 ± 6.8 ^*α*′*β*′*θ*bde′′^	95.7 ± 5.8	89.4 ± 10.5^*β*′′^	96.7 ± 4.8	95.0 ± 6.9						
Acacia honey	9–12	93.3 ± 7.0^*β*′′^	90.8 ± 7.0^*β*d^	93.8 ± 6.2	97.7 ± 3.7	97.3 ± 4.3						
s3												
No treatment	5–10	95.8 ± 6.8	96.0 ± 5.1	96.7 ± 7.5	99.2 ± 2.5	99.1 ± 2.8						
SSD	8–11	99.2 ± 1.8	99.8 ± 0.7	98.3 ± 4.0	98.2 ± 6.0	95.8 ± 11.8						
Manuka honey	7–11	90.4 ± 10.5^*β*^	88.2 ± 7.8^*αβ*′^	93.2 ± 13.2	96.6 ± 5.4	97.3 ± 5.8						
Acacia honey	6–12	93.1 ± 5.5	91.2 ± 7.0^*β*′d′′^	94.8 ± 5.5	97.8 ± 5.0	97.6 ± 5.8						
s4												
No treatment	5–10	98.1 ± 3.2	98.1 ± 4.3	97.1 ± 6.4	100.0 ± 0.0	98.5 ± 4.8						
SSD	8–11	100.0 ± 0.0	99.6 ± 1.2	100.0 ± 0.0	100.0 ± 0.0	100.0 ± 0.0						
Manuka honey	5–11	86.8 ± 9.1 ^*α*′*β*′*θ*′d′e^	90.9 ± 8.5^*β*^	96.5 ± 7.8	98.7 ± 3.0	97.8 ± 6.7						
Acacia honey	7–12	94.8 ± 4.4	90.4 ± 9.5^*β*d^	93.4 ± 8.1	98.9 ± 3.8	98.4 ± 4.4						

Values are expressed as mean ± SD, ANOVA, and Tukey-Kramer HSD.

*α*: *P* < 0.05 versus no treatment, *α*′: *P* < 0.01 versus no treatment, *α*′′: *P* < 0.1 versus no treatment, *β*: *P* < 0.05 versus SSD, *β*′: *P* < 0.01 versus SSD, *β*′′: *P* < 0.1 versus SSD, *γ*: *P* < 0.05 versus manuka honey, *γ*′: *P* < 0.01 versus manuka honey, *γ*′′: *P* < 0.1 versus manuka honey, *θ*: *P* < 0.05 versus acacia honey, *θ*′: *P* < 0.01 versus acacia honey, *θ*′′: *P* < 0.1 versus acacia honey, a: *P* < 0.05 versus 30 min, a′: *P* < 0.01 versus 30 min, a′′: *P* < 0.1 versus 30 min, b: *P* < 0.05 versus day 1, b′: *P* < 0.01 versus day 1, b′′: *P* < 0.1 versus day 1, c: *P* < 0.05 versus day 2, c′: *P* < 0.01 versus day 2, c′′: *P* < 0.1 versus day 2, d: *P* < 0.05 versus day 3, d′: *P* < 0.01 versus day 3, d′′: *P* < 0.1 versus day 3, e: *P* < 0.05 versus day 4, e′: *P* < 0.01 versus day 4, and e′′: *P* < 0.1 versus day 4.

**Table 3:** The proportions of apoptotic cells in each group.


Group	Epidermis (*n*)	30 min	Day 1	Day 2	Day 3	Day 4	Appendage (*n*)	30 min	Day 1	Day 2	Day 3	Day 4

c												
No treatment	5–9	0.9 ± 2.1	0.7 ± 1.5	0.0 ± 0.0	0.2 ± 0.5	0.0 ± 0.0	4–8	1.4 ± 2.1	0.0 ± 0.0	0.0 ± 0.0	0.3 ± 0.9	0.0 ± 0.0
SSD	7–11	0.0 ± 0.0	0.0 ± 0.0	0.0 ± 0.0	0.7 ± 2.2	0.0 ± 0.0	4–10	0.7 ± 2.0	0.5 ± 1.2	0.0 ± 0.0	0.0 ± 0.0	2.5 ± 4.8
Manuka honey	7–11	0.6 ± 1.4	0.2 ± 0.6	0.3 ± 0.7	0.0 ± 0.0	0.0 ± 0.0	6–11	0.8 ± 2.1	0.0 ± 0.0	1.6 ± 2.5	0.8 ± 2.2	0.6 ± 0.9
Acacia honey	4–10	0.3 ± 0.7	0.2 ± 0.6	0.0 ± 0.0	1.1 ± 2.7	0.0 ± 0.0	4–10	0.2 ± 0.5	1.0 ± 1.7	3.0 ± 4.4	1.5 ± 3.0	0.0 ± 0.0
s0												
No treatment	6–9	0.6 ± 1.1	2.1 ± 3.3	2.6 ± 4.1	0.6 ± 1.7	1.5 ± 2.4	5–9	1.9 ± 3.5	3.5 ± 4.8	1.0 ± 1.7	0.0 ± 0.0	1.0 ± 3.0
SSD	7–11	1.4 ± 1.8	1.7 ± 3.0	0.0 ± 0.0	0.0 ± 0.0	1.0 ± 1.5	5–11	1.1 ± 1.7	0.9 ± 2.2	0.6 ± 1.5	3.4 ± 6.3	4.2 ± 4.2
Manuka honey	6–11	0.0 ± 0.0	0.6 ± 1.8	0.0 ± 0.0	0.9 ± 2.6	0.0 ± 0.0	7–9	1.2 ± 2.4	1.5 ± 2.6	0.0 ± 0.0^*θ*^	3.3 ± 4.0	1.6 ± 2.7
Acacia honey	7–10	0.9 ± 1.7	0.4 ± 1.3	1.5 ± 3.1	0.9 ± 1.9	0.6 ± 1.1	4–10	0.5 ± 1.2	1.4 ± 2.0	3.3 ± 3.4	1.7 ± 3.5	0.0 ± 0.0
s1												
No treatment	5–9	0.5 ± 0.9	1.8 ± 3.2	1.7 ± 3.7	0.3 ± 0.8	0.1 ± 0.4 ^*γ*′′^	4–10	1.4 ± 2.2	4.3 ± 5.8	0.7 ± 1.8	1.9 ± 5.2	0.4 ± 0.9
SSD	7–11	0.6 ± 1.2	1.9 ± 2.4	0.2 ± 0.5^b^	0.3 ± 1.1^b′′^	0.2 ± 0.6^b′′^	8–11	0.0 ± 0.0^e^	1.5 ± 2.5	2.9 ± 3.4	2.7 ± 2.9	4.9 ± 5.7
Manuka honey	7–11	0.0 ± 0.0	2.8 ± 4.0	1.0 ± 2.1	0.2 ± 0.6	2.3 ± 3.2	7–11	0.8 ± 2.1	3.4 ± 3.1	2.5 ± 3.7	1.7 ± 4.1	3.3 ± 4.1
Acacia honey	5–10	1.3 ± 1.8	3.5 ± 5.5	0.7 ± 1.1	0.6 ± 1.1	1.1 ± 2.4	6–10	1.1 ± 2.3	1.4 ± 2.3	3.0 ± 6.4	4.0 ± 6.5	1.9 ± 3.0
s2												
No treatment	5–9	1.2 ± 1.5	1.8 ± 2.0	1.5 ± 1.7	1.5 ± 2.8	1.9 ± 2.8	4–10	1.6 ± 3.0	0.8 ± 1.9	0.3 ± 0.7	1.6 ± 3.0	3.1 ± 5.1
SSD	7–11	1.0 ± 2.0	0.4 ± 1.0	0.5 ± 1.2	0.0 ± 0.0	0.5 ± 1.0	8–11	2.0 ± 2.7	1.7 ± 3.8	0.6 ± 1.1	1.8 ± 2.0	3.0 ± 2.9
Manuka honey	7–11	0.6 ± 0.8	3.2 ± 4.7	0.0 ± 0.0^b′′^	1.0 ± 1.7	1.0 ± 1.7	7–11	0.5 ± 1.5	2.1 ± 4.4	1.3 ± 2.1	2.0 ± 2.9	1.9 ± 2.8
Acacia honey	7–10	0.8 ± 1.0	1.3 ± 2.5	0.7 ± 1.5	0.7 ± 1.2	0.8 ± 1.6	6–10	0.5 ± 1.5	1.7 ± 2.2	2.1 ± 4.9	2.2 ± 3.3	1.6 ± 3.8
s3												
No treatment	5–9	0.6 ± 1.7	0.7 ± 1.8	0.9 ± 1.2	1.5 ± 1.9	1.8 ± 3.0	4–11	0.6 ± 1.7	1.2 ± 1.6	2.5 ± 4.3	0.4 ± 1.0	0.6 ± 1.5
SSD	7–11	0.5 ± 1.5	0.2 ± 0.8	0.4 ± 1.3	0.0 ± 0.0	0.0 ± 0.0	8–11	1.7 ± 1.9	0.4 ± 0.7	0.5 ± 1.6	1.1 ± 1.9	1.0 ± 2.0
Manuka honey	7–11	0.0 ± 0.0	1.5 ± 4.4	0.8 ± 1.6	1.2 ± 1.7	0.6 ± 1.8	7–11	1.8 ± 3.8	0.2 ± 0.6	1.4 ± 1.6	1.1 ± 3.5	0.3 ± 0.8
Acacia honey	7–10	1.0 ± 1.2	0.5 ± 0.9	1.1 ± 2.2	0.3 ± 1.1	0.6 ± 1.0	6–10	0.9 ± 1.5	0.2 ± 0.4	1.2 ± 2.1	1.3 ± 2.2	0.5 ± 1.3
s4												
No treatment	5–9	1.9 ± 1.9	1.9 ± 1.7	1.8 ± 2.1	2.7 ± 3.1	1.5 ± 3.5	4–11	0.2 ± 0.5	1.5 ± 1.8	0.6 ± 1.5	1.9 ± 3.2	0.5 ± 1.6
SSD	7–11	1.4 ± 2.0	0.7 ± 1.3	1.1 ± 1.5	0.3 ± 1.0^*α*′^	0.2 ± 0.6	8–11	1.5 ± 4.5	1.3 ± 2.6	1.1 ± 1.8	0.6 ± 2.0	2.1 ± 3.2
Manuka honey	7–11	0.4 ± 0.7	0.1 ± 0.4^*α*′^	0.5 ± 0.9	0.6 ± 1.3^*α*′′^	0.6 ± 1.1	7–11	1.3 ± 3.1	0.7 ± 1.7	1.0 ± 2.7	0.0 ± 0.0	0.4 ± 1.1
Acacia honey	7–10	1.3 ± 1.3	0.0 ± 0.0^*α*′a′′^	0.4 ± 0.9	0.8 ± 1.5	0.2 ± 0.4	6–10	0.2 ± 0.6	0.9 ± 1.4	1.6 ± 2.2	1.2 ± 2.2	1.1 ± 1.8

Group	Dermis (*n*)	30 min	Day 1	Day 2	Day 3	Day 4	Endothelial cell (*n*)	30 min	Day 1	Day 2	Day 3	Day 4

c												
No treatment	6–11	0.6 ± 1.5	1.7 ± 1.3	0.7 ± 1.8	1.8 ± 4.1	1.1 ± 2.4	5–8	0.0 ± 0.0	0.0 ± 0.0	0.0 ± 0.0	0.0 ± 0.0	0.0 ± 0.0
SSD	8–11	1.6 ± 2.6	0.2 ± 0.5	0.0 ± 0.0	0.7 ± 2.1	0.6 ± 1.6	8–11	0.0 ± 0.0	1.8 ± 6.0	0.0 ± 0.0	0.0 ± 0.0	0.0 ± 0.0
Manuka honey	7–11	1.1 ± 1.4	1.0 ± 2.2	0.2 ± 0.7	1.8 ± 2.8	1.3 ± 2.6	7–9	0.0 ± 0.0	0.0 ± 0.0	0.0 ± 0.0	0.0 ± 0.0	0.0 ± 0.0
Acacia honey	6–10	1.1 ± 2.1	0.2 ± 0.6	0.4 ± 1.3	0.5 ± 1.4	0.0 ± 0.0	6–10	0.0 ± 0.0	3.0 ± 5.7	3.2 ± 8.4	5.6 ± 16.7	0.0 ± 0.0
s0												
No treatment	6–11	2.3 ± 3.6	1.7 ± 2.6	2.8 ± 3.0	2.0 ± 5.2	0.7 ± 2.2	5–8	0.0 ± 0.0	1.9 ± 4.5	10.4 ± 14.1	4.4 ± 8.0	0.0 ± 0.0
SSD	8–11	3.4 ± 3.1	5.0 ± 3.7	1.1 ± 1.6^b^	1.8 ± 2.2	2.5 ± 3.4	8–10	0.0 ± 0.0	1.7 ± 5.3	0.0 ± 0.0^*α*′′^	1.0 ± 3.1	0.0 ± 0.0
Manuka honey	7–11	1.0 ± 1.3	2.4 ± 3.6	2.2 ± 2.7	1.3 ± 3.5	0.4 ± 1.2	6–10	0.0 ± 0.0	1.1 ± 3.5	1.4 ± 3.9	0.0 ± 0.0	0.0 ± 0.0
Acacia honey	6–10	0.8 ± 1.3	2.8 ± 2.7	0.0 ± 0.0^*α*bd′′^	2.3 ± 2.7	0.2 ± 0.5^b′′^	6–10	0.0 ± 0.0	2.1 ± 5.9	4.1 ± 8.1	0.0 ± 0.0	0.0 ± 0.0
s1												
No treatment	6–11	0.7 ± 1.4	3.3 ± 3.0	1.7 ± 1.1	1.2 ± 1.6	1.5 ± 2.1	5–11	0.0 ± 0.0	0.0 ± 0.0	0.0 ± 0.0	0.0 ± 0.0	0.0 ± 0.0
SSD	8–11	2.9 ± 3.7	2.3 ± 3.1	1.0 ± 1.1	0.7 ± 1.9	2.8 ± 2.3	8–11	0.0 ± 0.0	0.0 ± 0.0	0.0 ± 0.0	0.0 ± 0.0	2.1 ± 5.9
Manuka honey	7–11	2.5 ± 2.2	2.7 ± 1.9	1.4 ± 2.1	2.5 ± 3.5	1.6 ± 4.6	6–11	0.0 ± 0.0	0.0 ± 0.0	5.4 ± 15.2	0.0 ± 0.0	0.0 ± 0.0
Acacia honey	6–10	1.1 ± 1.2	2.6 ± 3.1	1.6 ± 1.6	0.7 ± 1.0	0.5 ± 0.9	7–10	0.0 ± 0.0	0.0 ± 0.0	1.4 ± 4.5	0.0 ± 0.0	0.0 ± 0.0
s2												
No treatment	6–11	1.1 ± 1.2	2.2 ± 2.9	1.2 ± 1.7	1.8 ± 3.3	2.7 ± 5.2	5–9	0.0 ± 0.0	0.0 ± 0.0	2.7 ± 6.1	0.0 ± 0.0	0.0 ± 0.0
SSD	*n*8–11	2.1 ± 2.2	1.3 ± 1.8	1.1 ± 1.4	1.3 ± 1.6	1.6 ± 1.3	8–11	0.6 ± 1.7	0.0 ± 0.0	0.0 ± 0.0	0.0 ± 0.0	0.0 ± 0.0
Manuka honey	7–11	1.3 ± 1.7	2.8 ± 2.4	1.2 ± 1.7	0.6 ± 2.0	2.7 ± 2.4	7–11	0.0 ± 0.0	0.0 ± 0.0	0.0 ± 0.0	0.0 ± 0.0	0.0 ± 0.0
Acacia honey	6–10	1.2 ± 1.6	1.0 ± 0.9	1.8 ± 1.5	1.1 ± 1.6	1.6 ± 1.0	5–10	0.0 ± 0.0	4.2 ± 11.8	0.0 ± 0.0	0.0 ± 0.0	0.0 ± 0.0
s3												
No treatment	6–11	0.8 ± 1.3	2.0 ± 3.2	1.3 ± 1.9	1.5 ± 1.6	1.7 ± 1.5	4–7	0.0 ± 0.0	3.3 ± 7.5	4.2 ± 8.3	0.0 ± 0.0	0.0 ± 0.0
SSD	8–11	1.5 ± 1.7	1.3 ± 1.6	0.9 ± 1.6	1.4 ± 1.5	1.8 ± 2.0	7–11	0.0 ± 0.0	0.0 ± 0.0	0.0 ± 0.0^*α*′′^	0.0 ± 0.0	0.0 ± 0.0
Manuka honey	7–11	1.2 ± 1.8	1.7 ± 2.5	1.4 ± 2.4	0.8 ± 1.4	2.5 ± 3.9	4–11	0.0 ± 0.0	0.0 ± 0.0	0.0 ± 0.0^*α*′′^	0.0 ± 0.0	0.0 ± 0.0
Acacia honey	6–10	1.6 ± 2.4	0.6 ± 0.9	0.7 ± 1.2	0.9 ± 1.0	1.6 ± 2.3	5–10	1.9 ± 3.8	0.0 ± 0.0	0.0 ± 0.0^*α*′′^	0.0 ± 0.0	0.0 ± 0.0
s4												
No treatment	6–11	1.5 ± 1.5	1.7 ± 2.8	1.4 ± 1.5	1.1 ± 1.6	1.6 ± 2.3	4–7	0.0 ± 0.0	0.0 ± 0.0	3.1 ± 6.3	0.0 ± 0.0	0.0 ± 0.0
SSD	8–11	1.3 ± 2.1	1.8 ± 1.9	1.7 ± 2.7	1.9 ± 2.4	1.9 ± 2.3	8–11	0.0 ± 0.0	0.5 ± 1.7	0.0 ± 0.0	0.0 ± 0.0	0.0 ± 0.0
Manuka honey	7–11	0.2 ± 0.6	1.9 ± 2.1	2.1 ± 1.9	1.1 ± 2.3	1.6 ± 2.3	4–10	0.0 ± 0.0	0.0 ± 0.0	0.0 ± 0.0	0.0 ± 0.0	0.0 ± 0.0
Acacia honey	6–10	0.8 ± 1.3	1.5 ± 1.6	1.5 ± 1.8	1.2 ± 1.2	0.9 ± 1.0	7–9	0.0 ± 0.0	0.0 ± 0.0	1.3 ± 4.0	0.0 ± 0.0	0.0 ± 0.0

Group	Adipose (*n*)	30 min	Day 1	Day 2	Day 3	Day 4	Overall (*n*)	30 min	Day 1	Day 2	Day 3	Day 4

c												
No treatment	6–11	0.0 ± 0.0	0.0 ± 0.0	0.0 ± 0.0	4.1 ± 10.8	0.0 ± 0.0	6–11	0.7 ± 0.7	1.6 ± 0.9	1.8 ± 1.3	1.2 ± 1.7	0.9 ± 1.0
SSD	8–11	0.0 ± 0.0	5.0 ± 9.2	0.0 ± 0.0	0.0 ± 0.0	0.0 ± 0.0	9–11	1.0 ± 0.7	1.3 ± 0.7	0.5 ± 0.3^*α*′b′′^	0.8 ± 0.7	1.0 ± 0.6
Manuka honey	7–11	0.0 ± 0.0	0.0 ± 0.0	0.0 ± 0.0	0.0 ± 0.0	0.0 ± 0.0	7–11	0.6 ± 0.3	1.1 ± 0.6	0.9 ± 0.6	0.7 ± 0.5	0.7 ± 0.3
Acacia honey	7–10	0.5 ± 1.5	0.3 ± 0.8	0.7 ± 2.3	0.0 ± 0.0	2.9 ± 7.6	7–11	0.7 ± 0.4	1.5 ± 1.3	1.3 ± 0.5	0.9 ± 0.8	0.7 ± 0.3
s0												
No treatment	6–11	0.0 ± 0.0	3.6 ± 6.1	3.1 ± 5.4	1.3 ± 3.4	0.0 ± 0.0	Normal skin (*n*)					
SSD	8–11	0.0 ± 0.0	2.6 ± 5.9	0.0 ± 0.0	0.0 ± 0.0	0.0 ± 0.0	6	Epidermis	0.7 ± 1.3			
Manuka honey	7–11	0.0 ± 0.0	1.3 ± 4.0	1.0 ± 2.7	0.0 ± 0.0	0.0 ± 0.0	6	Appendage	0.7 ± 1.3			
Acacia honey	6–10	0.0 ± 0.0	0.6 ± 1.8	0.0 ± 0.0	0.0 ± 0.0	1.0 ± 2.4	6	Dermis	0.3 ± 0.5			
s1												
No treatment	6–11	0.0 ± 0.0	0.0 ± 0.0	2.3 ± 3.6	0.0 ± 0.0	1.8 ± 6.0	6	Blood vessel	0.0 ± 0.0			
SSD	8–11	0.4 ± 1.1	3.7 ± 6.7	2.1 ± 4.6	1.1 ± 3.5	0.0 ± 0.0	6	Adipose	0.0 ± 0.0			
Manuka honey	7–11	0.0 ± 0.0	1.1 ± 3.5	0.0 ± 0.0	0.0 ± 0.0	0.0 ± 0.0	6	All	0.4 ± 0.7			
Acacia honey	7–10	0.9 ± 2.1	7.5 ± 16.0	2.6 ± 5.3	1.3 ± 2.7	0.0 ± 0.0						
s2												
No treatment	5–9	0.0 ± 0.0	2.2 ± 3.4	2.1 ± 3.1	2.2 ± 5.0	0.9 ± 2.6						
SSD	4–11	1.3 ± 3.0	0.5 ± 1.7	0.0 ± 0.0 ^*α*^	0.0 ± 0.0	0.0 ± 0.0						
Manuka honey	7–11	0.0 ± 0.0	1.3 ± 4.3	0.0 ± 0.0 ^*α*′′^	0.0 ± 0.0	0.0 ± 0.0						
Acacia honey	5–9	0.0 ± 0.0	1.3 ± 2.5	0.0 ± 0.0 ^*α*^	0.0 ± 0.0	0.0 ± 0.0						
s3												
No treatment	4–8	0.8 ± 2.4	0.8 ± 1.9	2.3 ± 2.7	0.0 ± 0.0	0.4 ± 1.3						
SSD	4–11	0.5 ± 1.4	0.0 ± 0.0 ^*θ*′′^	0.0 ± 0.0^*α*′′^	0.0 ± 0.0	0.0 ± 0.0						
Manuka honey	5–11	1.5 ± 3.4	0.0 ± 0.0^*θ*′′^	0.0 ± 0.0^*α*′′^	0.0 ± 0.0	0.0 ± 0.0						
Acacia honey	4–9	0.8 ± 1.7	1.7 ± 2.8	1.1 ± 2.0	0.0 ± 0.0	0.7 ± 1.6						
s4												
No treatment	4–9	0.0 ± 0.0	1.1 ± 2.6	6.3 ± 12.5	0.0 ± 0.0	0.0 ± 0.0						
SSD	4–11	1.0 ± 3.0	0.5 ± 1.7	0.0 ± 0.0	0.0 ± 0.0	0.0 ± 0.0						
Manuka honey	7–10	0.0 ± 0.0	0.0 ± 0.0	0.0 ± 0.0	0.0 ± 0.0	0.0 ± 0.0						
Acacia honey	4–9	0.0 ± 0.0	0.0 ± 0.0	0.3 ± 1.0	0.0 ± 0.0	1.8 ± 2.9						

Values are expressed as mean ± SD, ANOVA, and Tukey-Kramer HSD.

*α*: *P* < 0.05 versus no treatment, *α*′: *P* < 0.01 versus no treatment, *α*′′: *P* < 0.1 versus no treatment, *γ*′′: *P* < 0.1 versus manuka honey, *θ*: *P* < 0.05 versus acacia honey, *θ*′′: *P* < 0.1 versus Acacia honey, a′′: *P* < 0.1 versus 30 min, b: *P* < 0.05 versus day 1, b′′: *P* < 0.1 versus day 1, d′′: *P* < 0.1 versus day 3, and e: *P* < 0.05 versus day 4.

**Table 4:** The number of neutrophils and macrophages in each group.


Group	Neutrophil						Macrophage					
	Dermis (*n*)	30 min	Day 1	Day 2	Day 3	Day 4	Dermis (*n*)	30 min	Day 1	Day 2	Day 3	Day 4

c												
No treatment	7–12	52.5 ± 41.5	37.8 ± 50.7^*β*′^	464.6 ± 774.9	549.8 ± 571.6	381.2 ± 506.3	5–11	133.4 ± 154.0	46.7 ± 58.7	28.0 ± 47.2	48.2 ± 56.6	66.5 ± 68.6
SSD	7–11	34.2 ± 46.9^b′c′d′e′^	263.0 ± 126.3	378.6 ± 107.5	223.5 ± 88.6^*α*′′c^	242.3 ± 149.8^c′′^	8–11	42.0 ± 41.5^*α*′′^	53.8 ± 65.4	4.7 ± 10.5^b′′^	29.7 ± 39.6	25.3 ± 32.1
Manuka honey	9–11	8.5 ± 14.5^bc′d′^	155.6 ± 116.4	202.3 ± 167.8	222.5 ± 115.5^*α*′′^	134.9 ± 122.5	7–12	26.5 ± 38.8 ^*α*^	22.0 ± 50.3	44.5 ± 56.4	41.0 ± 41.9	3.9 ± 11.0^*α*^
Acacia honey	8–10	32.9 ± 51.9^e^	74.3 ± 73.3^*β*′^	59.1 ± 41.4^e′′^	113.6 ± 106.6^*α*^	208.1 ± 213.8	9–11	37.3 ± 43.5^*α*′′^	66.9 ± 43.4	41.0 ± 28.9	24.0 ± 24.5^b^	22.5 ± 19.2^b^
s0												
No treatment	7–12	25.3 ± 26.2^c′′de^	393.5 ± 288.4	455.1 ± 474.1	517.0 ± 351.6	470.7 ± 317.5	5–11	113.4 ± 147.8	74.3 ± 85.5	80.9 ± 89.7	66.9 ± 70.4	116.0 ± 113.1
SSD	7–11	31.1 ± 51.3^b′c′d′e′^	269.7 ± 226.3	364.8 ± 98.4	322.5 ± 145.8	375.7 ± 147.7	8–11	56.0 ± 58.3	43.9 ± 63.7	31.1 ± 20.7	79.2 ± 55.4	54.5 ± 31.1
Manuka honey	9–11	21.2 ± 20.0^b′cd′e^	261.4 ± 140.5	224.1 ± 160.0	255.2 ± 134.9^*α*′′^	221.3 ± 217.5	7–12	29.6 ± 53.1	27.2 ± 47.4	35.6 ± 40.9	42.4 ± 28.8	27.2 ± 37.0^*α*^
Acacia honey	8–10	17.3 ± 31.6^de′^	73.9 ± 46.1^*α*e^	118.3 ± 90.2^*α*e′′^	311.2 ± 231.7	387.1 ± 378.3	9–11	28.0 ± 35.0^b′^	87.1 ± 52.0	48.1 ± 27.4	31.1 ± 42.9^b^	27.7 ± 26.7^*α*b^
s1												
No treatment	7–12	31.1 ± 20.4^e′′^	173.4 ± 290.0	31.1 ± 39.2^*β*′^	285.3 ± 328.8	333.3 ± 325.6	5–11	86.7 ± 60.2	60.5 ± 75.2	127.6 ± 62.6	37.3 ± 45.3	114.6 ± 115.0
SSD	7–11	21.8 ± 42.9^c′d′^	163.4 ± 226.8	401.1 ± 213.3	389.0 ± 367.8	206.7 ± 111.0	8–11	7.8 ± 13.2^*α*′d′′e′′^	63.7 ± 58.0	38.9 ± 55.0^*α*′′^	94.8 ± 116.3	101.1 ± 70.6
Manuka honey	9–11	15.6 ± 16.4^d′^	136.9 ± 112.4	107.4 ± 158.3^*β*′d′′^	396.8 ± 452.3	179.8 ± 170.0	7–12	9.3 ± 16.7^*α*′^	46.7 ± 80.7	73.4 ± 82.7	36.8 ± 29.7	25.3 ± 24.9^*α*′′^
Acacia honey	8–10	38.0 ± 52.3^e^	48.4 ± 55.8^e^	119.8 ± 215.1^*β*′′^	126.0 ± 114.2	320.9 ± 345.4	9–11	14.0 ± 20.0^*α*′bc′^	71.6 ± 39.6	79.2 ± 46.9	34.0 ± 49.6^c′′^	41.5 ± 38.1
s2												
No treatment	7–12	29.2 ± 30.5	71.1 ± 70.1	15.6 ± 23.8	57.1 ± 103.5^*β*′′^	38.9 ± 59.2	5–11	26.7 ± 34.5	57.1 ± 87.7	99.6 ± 76.7	34.2 ± 63.0	56.6 ± 66.5^*β*′^
SSD	7–11	15.6 ± 12.7^d′e′′^	20.2 ± 19.5^d′^	45.0 ± 54.2^d^	168.3 ± 160.5	128.9 ± 78.7	8–11	7.8 ± 16.8^de′^	17.0 ± 24.6^d′′e′^	29.6 ± 38.4^e′^	110.3 ± 118.8	178.9 ± 131.0
Manuka honey	9–11	26.5 ± 36.7	94.9 ± 146.8	24.9 ± 42.9	14.0 ± 15.5^*β*′^	46.7 ± 83.4	7–12	4.7 ± 7.5^b′′^	59.6 ± 75.3	28.9 ± 42.5	52.3 ± 44.2	33.1 ± 36.7^*β*′^
Acacia honey	8–10	10.4 ± 20.6^e^	24.2 ± 56.2^e^	3.1 ± 9.8^*β*′′e^	21.8 ± 32.1^*β*′e^	214.0 ± 304.6	9–11	9.3 ± 16.7	59.1 ± 60.8	63.7 ± 62.4	31.1 ± 33.4^*β*′′^	27.7 ± 30.9^*β*′^
s3												
No treatment	6–12	17.8 ± 22.8	82.2 ± 54.4	15.6 ± 17.0	95.1 ± 250.7	25.9 ± 26.0	5–11	26.7 ± 29.4	24.2 ± 41.3	37.3 ± 35.8	42.0 ± 55.9	55.2 ± 72.8^*β*′^
SSD	7–11	37.3 ± 55.5^d′′^	29.6 ± 43.1^d^	53.6 ± 58.8	137.2 ± 138.0	128.9 ± 60.7	8–11	9.3 ± 13.1^d′′e′^	15.6 ± 32.6^e′^	20.2 ± 53.9^e′^	93.4 ± 89.1	163.4 ± 129.9
Manuka honey	9–11	31.1 ± 51.9	46.7 ± 74.8	23.3 ± 37.6	48.2 ± 90.6	77.8 ± 79.0	7–12	6.2 ± 8.0^*α*′′^	54.5 ± 87.4	31.1 ± 44.9	48.1 ± 39.0	17.5 ± 32.7^*β*′^
Acacia honey	8–10	6.9 ± 11.3	10.4 ± 11.0^*α*^	3.1 ± 6.6^*β*^	4.7 ± 7.5	75.9 ± 202.1	9–11	7.8 ± 13.2^c′′^	46.7 ± 51.9	49.5 ± 56.9	26.9 ± 35.5^*β*′′^	25.9 ± 25.8^*β*′^
s4												
No treatment	6–12	20.7 ± 18.8	91.1 ± 73.2	10.4 ± 18.8	70.9 ± 178.8	45.4 ± 80.0	5–11	62.2 ± 74.1	24.2 ± 39.8	15.6 ± 27.0	56.0 ± 88.1	69.3 ± 82.7
SSD	7–11	28.0 ± 17.7	18.7 ± 26.2^*α*′^	58.8 ± 101.7	86.3 ± 86.7	106.7 ± 82.1	8–11	4.7 ± 10.5^*α*d′′e′^	17.0 ± 27.4^e^	18.7 ± 43.9^e^	70.7 ± 64.7	107.0 ± 108.3
Manuka honey	9–11	10.9 ± 12.8	10.9 ± 19.5^*α*′^	4.7 ± 10.5^e′′^	23.3 ± 32.2	48.4 ± 68.0	7–12	12.4 ± 29.2 ^*α*^	24.6 ± 45.2	17.8 ± 34.1	22.6 ± 25.4	17.5 ± 24.2
Acacia honey	8–10	12.1 ± 26.7	20.7 ± 37.3^*α*′^	3.1 ± 9.8	43.6 ± 79.3	58.8 ± 148.2	7–11	6.2 ± 13.1^*α*b′′^	43.6 ± 44.5	28.3 ± 39.3	22.6 ± 33.6	12.1 ± 17.0^*β*^

Group	Hypodermis (*n*)	30 min	Day 1	Day 2	Day 3	Day 4	Hypodermis (*n*)	30 min	Day 1	Day 2	Day 3	Day 4

c												
No treatment	7–12	21.4 ± 18.5	306.8 ± 234.0	126.7 ± 126.9	527.1 ± 556.3	420.1 ± 604.5	5–11	77.8 ± 107.4	126.2 ± 152.9	115.1 ± 128.1	84.0 ± 114.4	137.2 ± 142.1
SSD	7–11	62.2 ± 94.8^b′′c′′^	266.1 ± 218.9	275.4 ± 213.1	171.2 ± 119.9	224.5 ± 224.6	9–11	46.7 ± 24.3	65.1 ± 52.5	94.9 ± 64.6	86.3 ± 66.5	74.3 ± 69.9
Manuka honey	9–11	8.5 ± 10.7^*β*′′^	312.8 ± 220.8	185.0 ± 233.9	326.8 ± 301.8	311.2 ± 665.0	8–12	20.2 ± 18.0^d′^	44.1 ± 49.1^d′^	60.3 ± 59.7^d′′^	138.6 ± 103.1	58.4 ± 54.4^d′′^
Acacia honey	9–12	17.3 ± 25.1 ^d^	143.5 ± 180.3	68.5 ± 67.3^*β*′′*d*′′^	329.9 ± 406.5	114.1 ± 169.5	9–11	34.2 ± 28.2	71.6 ± 60.6	83.5 ± 45.8	59.4 ± 62.9	38.0 ± 28.2^*α*′′^
s0												
No treatment	7–12	9.7 ± 11.6^d′^	275.6 ± 164.1	301.5 ± 407.8	453.2 ± 325.8	273.6 ± 151.5	5–11	55.6 ± 58.8	124.5 ± 117.7	143.2 ± 94.9	79.4 ± 89.1	182.5 ± 147.3
SSD	7–11	71.6 ± 167.9^c^	168.3 ± 164.4^*γ*^	336.1 ± 264.9	164.1 ± 80.4	171.2 ± 150.0	9–11	34.2 ± 31.8^d^	63.7 ± 51.4	77.8 ± 26.4	121.7 ± 90.7	84.7 ± 76.3
Manuka honey	9–11	12.7 ± 18.2^b′d′′^	395.2 ± 202.4	138.3 ± 157.0	270.8 ± 369.9	150.4 ± 217.6	8–12	23.3 ± 32.2^d′^	55.8 ± 51.2^d′^	58.4 ± 56.9^*α*d^	147.1 ± 99.5	48.6 ± 45.1^*α*d^
Acacia honey	9–12	22.5 ± 22.2 ^d′′^	126.2 ± 115.5 ^*γ*′^	96.5 ± 136.4	256.7 ± 322.6	131.0 ± 217.6	9–11	32.7 ± 18.6^bcd′′^	94.9 ± 40.5	87.7 ± 43.1	86.3 ± 70.7	45.0 ± 21.2^*α*^
s1												
No treatment	7–12	46.7 ± 39.0	146.7 ± 155.3^*γ*′′^	44.7 ± 66.2^*β*^	326.8 ± 416.0	334.6 ± 360.7	5–11	33.3 ± 39.6	126.2 ± 97.6	149.4 ± 103.6	113.6 ± 129.4	134.4 ± 123.4
SSD	7–11	15.6 ± 16.4^*α*c′^	175.8 ± 198.5^*γ*′′^	380.4 ± 360.4	207.9 ± 174.3	122.3 ± 135.5	9–11	38.9 ± 32.2	131.6 ± 101.6	118.3 ± 110.1	111.8 ± 89.6	83.0 ± 67.8
Manuka honey	9–11	21.8 ± 16.7^b^	393.7 ± 277.1	77.8 ± 79.7^*β*^	127.6 ± 116.2	278.4 ± 574.9	8–12	12.4 ± 23.0^d′^	80.4 ± 77.6	54.5 ± 78.5	121.7 ± 64.8	70.0 ± 50.6
Acacia honey	9–12	10.4 ± 11.0^*α*^	88.2 ± 103.5^*γ*^	116.7 ± 253.5^*β*′′^	51.3 ± 42.2^*α*′′^	190.6 ± 492.1	9–11	37.3 ± 39.6^b^	136.9 ± 71.0	106.1 ± 75.9	70.7 ± 79.1	50.1 ± 40.3^b^
s2												
No treatment	7–12	23.3 ± 26.3	51.1 ± 33.3^*γ*^	15.6 ± 18.0	55.6 ± 79.8	210.1 ± 628.7	5–11	37.8 ± 65.3	81.3 ± 93.6	108.9 ± 87.3	96.5 ± 135.0	124.5 ± 156.4
SSD	7–11	29.6 ± 33.2	45.1 ± 52.6^*γ*^	76.2 ± 99.1	55.2 ± 43.7	113.4 ± 185.8	9–11	31.1 ± 30.2^b′e^	149.9 ± 61.6	85.6 ± 70.5	99.0 ± 74.4	124.5 ± 66.9
Manuka honey	9–11	21.8 ± 16.7^b^	331.4 ± 369.0	18.7 ± 39.4^b^	45.1 ± 121.3^b^	115.8 ± 248.3	8–12	9.3 ± 10.9b	119.3 ± 131.6	48.6 ± 78.7	93.4 ± 82.3	52.5 ± 39.0
Acacia honey	9–12	17.3 ± 18.2	15.6 ± 17.4^*γ*′^	7.8 ± 15.1^*β*′′^	26.5 ± 45.8	164.7 ± 353.0	9–11	18.7 ± 17.7^b′^	135.4 ± 119.9	89.1 ± 81.8	46.7 ± 58.6^b′′^	67.4 ± 56.6
s3												
No treatment	7–12	11.1 ± 11.8	77.8 ± 107.8	33.3 ± 75.4	184.8 ± 479.0	27.2 ± 70.9	5–11	15.6 ± 29.8	65.7 ± 81.1	18.7 ± 25.6	79.4 ± 98.3	75.0 ± 89.1
SSD	7–11	31.1 ± 38.8	21.8 ± 19.7	31.1 ± 40.8	59.4 ± 67.8	48.9 ± 37.5	9–11	20.2 ± 22.1	70.7 ± 54.5	43.6 ± 78.3	46.7 ± 35.5	65.7 ± 59.1
Manuka honey	9–11	29.6 ± 46.1	23.3 ± 33.8	0.0 ± 0.0	10.9 ± 29.4	22.5 ± 36.6	8–11	21.8 ± 30.4^b′^	158.2 ± 149.8	42.8 ± 77.9^b^	62.2 ± 62.6	25.3 ± 24.9^b^
Acacia honey	9–12	25.9 ± 32.1	15.6 ± 13.5^*α*′′^	0.0 ± 0.0	15.6 ± 18.0	73.6 ± 172.2	9–11	21.8 ± 25.6^b′^	108.9 ± 80.4	55.2 ± 67.6	19.8 ± 21.0^b′^	46.7 ± 43.3^b′′^
s4												
No treatment	7–12	13.0 ± 11.7	17.8 ± 24.5	18.2 ± 31.8	194.5 ± 525.1	32.4 ± 70.0	5–11	73.4 ± 86.5	48.4 ± 73.2	0.0 ± .0.0^*θ*′′^	62.2 ± 100.8	84. ± 133.7
SSD	7–11	28.0 ± 29.2	12.4 ± 14.3	42.0 ± 97.3	31.1 ± 26.0	42.2 ± 33.3	9–11	24.9 ± 21.0	72.1 ± 75.0	17.1 ± 30.6	50.9 ± 29.6	76.0 ± 73.6
Manuka honey	9–11	6.2 ± 15.0	28.0 ± 26.2	7.8 ± 19.8	26.5 ± 33.7	24.2 ± 43.4	8–12	12.4 ± 20.5^*α*b′^	142.6 ± 130.2	23.3 ± 16.6^b′^	41.0 ± 48.8^b′^	23.3 ± 14.4^b′^
Acacia honey	9–12	8.6 ± 11.3	8.6 ± 11.3^*γ*′′^	6.2 ± 10.9	28.0 ± 56.2	49.3 ± 115.1	9–11	14.0 ± 20.0^*α*b′^	96.5 ± 72.5	34.0 ± 27.7^b′^	31.1 ± 29.5^b′^	29.4 ± 27.4^b′^

Group	Overall (*n*)	30 min	Day 1	Day 2	Day 3	Day 4	Overall (*n*)	30 min	Day 1	Day 2	Day 3	Day 4

No treatment	7–12	25.5 ± 13.2 ^de^	143.8 ± 83.8	111.5 ± 123.7	260.1 ± 240.0	216.1 ± 151.6	9–11	61.9 ± 45.8	71.6 ± 63.6	77.0 ± 50.7	66.7 ± 65.1	101.4 ± 88.8
SSD	7–11	33.8 ± 30.9^b′′c′d′e^	122.2 ± 61.3	190.6 ± 100.7	168.0 ± 87.6	159.3 ± 84.6	9–11	27.4 ± 16.7^*α*′′de^	63.7 ± 31.6	48.4 ± 27.9	87.9 ± 47.3	89.0 ± 60.3
Manuka honey	9–11	18.0 ± 12.8 ^b′de′′^	182.6 ± 85.3	82.7 ± 58.1^*β*^	147.3 ± 90.2	134.3 ± 164.0	8–12	15.7 ± 17.5^*α*′b′′d′′^	69.6 ± 64.9	41.8 ± 45.1	70.6 ± 43.5	33.6 ± 25.4^*α*′′^
Acacia honey	9–12	18.3 ± 19.3^e′′^	53.5 ± 43.2^*α*′′*γ*′^	50.2 ± 56.5^*β*′^	110.7 ± 76.7^*α*′′^	132.4 ± 175.7	9–11	21.8 ± 18.6^*α*b′c^	84.9 ± 47.1	63.8 ± 34.2	40.3 ± 38.1^b^	36.2 ± 19.8 ^*α*′′b^

Values are expressed as mean ± SD, ANOVA, and Tukey-Kramer HSD.

*α*: *P* < 0.05 versus no treatment, *α*′: *P* < 0.01 versus no treatment, *α*′′: *P* < 0.1 versus no treatment, *β*: *P* < 0.05 versus SSD, *β*′: *P* < 0.01 versus SSD, *β*′′: *P* < 0.1 versus SSD, *θ*′′: *P* < 0.1 versus acacia honey, b: *P* < 0.05 versus day 1, b′: *P* < 0.01 versus day 1, b′′: *P* < 0.1 versus day 1, c: *P* < 0.05 versus day 2, c′: *P* < 0.01 versus day 2, c′′: *P* < 0.1 versus day 2, d: *P* < 0.05 versus day 3, d′: *P* < 0.01 versus day 3, d′′: *P* < 0.1 versus day 3, e: *P* < 0.05 versus day 4, e′: *P* < 0.01 versus day 4, and e′′: *P* < 0.1 versus day 4.

**Table 5:** The level of serum TNF-*α* in each group.

	30 min	Day 1	Day 2	Day 3	Day 4
No treatment	8.8 ± 0.8^b^	12.3 ± 2.2	10.2 ± 1.0	11.0 ± 1.5	9.5 ± 0.8^γ′′b′′^
SSD	9.2 ± 1.2	16.6 ± 11.2	11.6 ± 1.2	10.2 ± 0.7	12.1 ± 2.2
Manuka honey	8.8 ± 0.7	10.4 ± 2.2	12.4 ± 4.2	10.5 ± 1.0	12.5 ± 1.5
Acacia honey	8.7 ± 0.7	11.4 ± 1.0^c′^	6.9 ± 2.9^γ′′^	11.4 ± 0.9^c′^	11.5 ± 1.0^c^

Values are expressed as mean ± SD, ANOVA, and Tukey-Kramer HSD. *n* = 3-4 in all groups.

*γ*′′: *P* < 0.1 versus manuka honey, b: *P* < 0.05 versus day 1, b′′: *P* < 0.1 versus day 1, c: *P* < 0.05 versus day 2, and c′: *P* < 0.01 versus day 2.

**Table 6:** The characteristics of each zone on burn wound.

	Types of burns	Zone of coagulation	Zone of stasis	Zone of hyperemia
Jackson [2]	Flame, scald (human)	White color macroscopically Nonpatent blood vessels Coagulation tissue	Change from red to white color macroscopically Loss of blood vessels and necrosis until days 3–7	Turn to red color until day 4 macroscopically Heal until day 7 Loss of epidermis, normal dermis, and patent blood vessels

Present research (H&E)	Scald (mice)	White color macroscopically Nonpatent blood vessels Coagulation and necrotic tissue	White color macroscopicallyLoss of epidermis around day 2 or 3, covered surface with eosinophilic like material, and formation of clot	Turn to red color around day 2 or 3 Retention of epidermis, normal dermis, and patent blood vessels

Present research (anti-HMGB1 antibody, day 4, and no treatment)	Scald (mice)	Almost 100% in all parameters	Epidermis: 93.0% Appendage: 89.3% Dermis: 94.68% Blood vessel: 96.2% Adipose tissue: 98.0%	Epidermis: 86.7% Appendage: 80.2% Dermis: 93.5% Blood vessel: 96.3% Adipose tissue: 97.5%

Present research (anti-HMGB1 antibody, unburned skin)	Unburned skin	Epidermis: 50.2% Appendage: 47.0% Dermis: 62.8% Blood vessel: 56.8% Adipose tissue: 57.2%		

## References

[1] Johnson RM, Richard R (2003). Partial-thickness burns: identification and management. *Advances in Skin & Wound Care*.

[2] Jackson DM (1953). The diagnosis of the depth of burning. *British Journal of Surgery*.

[3] Shupp JW, Nasabzadeh TJ, Rosenthal DS, Jordan MH, Fidler P, Jeng JC (2010). A review of the local pathophysiologic bases of burn wound progression. *Journal of Burn Care and Research*.

[4] Khoo Y-T, Halim AS, Singh K-KB, Mohamad N-A (2010, article 48). Wound contraction effects and antibacterial properties of Tualang honey on full-thickness burn wounds in rats in comparison to hydrofibre. *BMC Complementary and Alternative Medicine*.

[5] Agata KD, Ewa SS, Robert DW (2004). Efficiency assessment of antimicrobial activity of honey-balm on experimental burn wounds. *Bulletin of the Veterinary Institute in Pulawy*.

[6] Jull AB, Rodgers A, Walker N (2008). Honey as a topical treatment for wounds. *Cochrane Database of Systematic Reviews*.

[7] Nisbet HO, Nisbet C, Yarim M, Guler A, Ozak A (2010). Effects of three types of honey on cutaneous wound healing. *Wounds*.

[8] Hashemi B, Bayat A, Kazemei T, Azarpira N (2011). Comparison between topical honey and mafenide acetate in treatment of auricular burn. *The American Journal of Otolaryngology*.

[9] Song JJ, Salcido R (2011). Use of honey in wound care: an update. *Advances in Skin & Wound Care*.

[10] Molan PC (2001). Potential of honey in the treatment of wounds and burns. *The American Journal of Clinical Dermatology*.

[11] Li J, Chen J, Kirsner R (2007). Pathophysiology of acute wound healing. *Clinics in Dermatology*.

[12] Singh V, Devgan L, Bhat S, Milner SM (2007). The pathogenesis of burn wound conversion. *Annals of Plastic Surgery*.

[13] Horton JW (2003). Free radicals and lipid peroxidation mediated injury in burn trauma: the role of antioxidant therapy. *Toxicology*.

[14] Yoshikawa T (2011). Free radicals and medicine. *Journal of Kyoto Prefectural University of Medicine*.

[15] Singer AJ, McClain SA, Taira BR, Guerriero JL, Zong W (2008). Apoptosis and necrosis in the ischemic zone adjacent to third degree burns. *Academic Emergency Medicine*.

[16] Lanier ST, McClain SA, Lin F, Singer AJ, Clark RAF (2011). Spatiotemporal progression of cell death in the zone of ischemia surrounding burns. *Wound Repair and Regeneration*.

[17] Haryanto, Urai T, Mukai K, Suriadi, Sugama J, Nakatani T (2012). Effectiveness of Indonesian honey on the acceleration of cutaneous wound healing: an experimental study in mice. *Wounds*.

[18] Nakajima Y, Nakano Y, Fuwano S (2013). Effects of three types of Japanese honey on full-thickness wound in mice. *Evidence-Based Complementary and Alternative Medicine*.

[19] Working group in Japanese society for burn injuries (2009). *Guidelines for Burn Management*.

[20] Singer AJ, Berruti L, Thode HC, Mcclain SA (2000). Standardized burn model using a multiparametric histologic analysis of burn depth. *Academic Emergency Medicine*.

[21] Takamiya M, Saigusa K, Nakayashiki N, Aoki Y (2001). A histological study on the mechanism of epidermal nuclear elongation in electrical and burn injuries. *International Journal of Legal Medicine*.

[22] Papp A, Kiraly K, Härmä M, Lahtinen T, Uusaro A, Alhava E (2004). The progression of burn depth in experimental burns: a histological and methodological study. *Burns*.

[23] Meyerholz DK, Piester TL, Sokolich JC, Zamba GKD, Light TD (2009). Morphological parameters for assessment of burn severity in an acute burn injury rat model. *International Journal of Experimental Pathology*.

[24] Moore OA, Smith LA, Campbell F, Seers K, McQuay HJ, Moore RA (2001). Systematic review of the use of honey as a wound dressing. *BMC Complementary and Alternative Medicine*.

[25] Gates JL, Holloway GA (1992). A comparison of wound environments. *Ostomy/Wound Management*.

[26] Biglari B, Moghaddam A, Santos K, Blaser G, Büchler A, Jansen G (2013). Multicentre prospective observational study on professional wound care using honey (Medihoney). *International Wound Journal*.

